# Meta-analysis of smartphone applications targeting eating behaviour for weight loss

**DOI:** 10.1038/s41598-026-56407-7

**Published:** 2026-06-05

**Authors:** Kadri Arumäe, Neeme Ilves, Eno-Martin Lotman, Triin Viskus, Tõnu Esko, Priit Palta, Taavi Tillmann, Uku Vainik

**Affiliations:** 1 Institute of Psychology, University of Tartu, Näituse 2, 50409 Tartu, Estonia; 2 Institute of Family Medicine and Public Health, University of Tartu, Tartu, Estonia; 3 Cardiology Centre, North Estonia Medical Centre, Tallinn, Estonia; 4 Department of Health Technologies, Tallinn University of Technology, Tallinn, Estonia; 5 Department of Sustainable Development, North Estonia Medical Centre, Tallinn, Estonia; 6 Estonian Genome Centre, Institute of Genomics, University of Tartu, Tartu, Estonia; 7 Department of Neurology and Neurosurgery, McGill University, Montreal, Canada; 8 Activate Health, Tallinn, Estonia

**Keywords:** Weight loss, Smartphone applications, Three-level meta-analysis, Bias correction, Diseases, Health care, Medical research

## Abstract

Smartphone applications (“apps”) could be safe, cost-effective, and accessible tools for weight loss. Numerous weight-loss apps are available, but their longer-term efficacy is unclear. We conducted a meta-analysis of randomised controlled trials of weight loss apps that target eating behaviour in adults. We (a) compared the amount of weight lost in app-based interventions to other interventions and waitlists, and (b) estimated mean weight loss at six months and longer in app-based interventions. A database search identified 23 studies matching our criteria. We used three-level meta-analytic models and applied various bias-correction methods. More weight was lost in app-based interventions than on waitlists (mean difference 2.07 kg) and in non-app-based interventions (mean difference 1.64 kg) at six months. In app-based interventions, weight loss estimates ranged from 0.63 to 3.87 kg at six months; mean weight loss was 2.64 kg at longer follow-ups. We found no moderating effects of factors such as calorie counting, social support, involvement of medical workers, or automatic feedback reports. Smartphone apps could be cost-effective tools to facilitate weight loss and help maintain weight loss after surgery or drug treatments, but more studies on apps’ long-term effectiveness are needed.

**Supplementary Information:**

The online version contains supplementary material available at 10.1038/s41598-026-56407-7.

## Introduction

Excess body weight is a major risk factor for premature mortality and morbidity, accounting for four million deaths and loss of 120 million disease-adjusted life years worldwide per year^1^ and its contribution has been escalating^2^. Drastic action is necessary to limit its impact. Although substantial improvements could be achieved by modifying the food environment—for instance, by limiting the availability of hyperpalatable food—society-level changes take time and are difficult to implement^3^. Person-centered approaches are needed to help people currently at risk.

Standard weight-loss interventions fall into three classes: behavioural modification, drug treatments, and bariatric surgery. However, behavioural modification tends to be resource-intensive (e.g., requiring personal assistance of trained medical professionals), drug treatments can have unwanted side effects, and surgery is invasive and carries a risk of complications. Smartphone applications (“apps”), increasingly used to facilitate weight loss, are a promising alternative that circumvents these issues. App-based interventions target the same behaviours as traditional behavioural modification, such as diet, physical activity, and/or other aspects of lifestyle, but are scalable to large populations and considerably cut down on financial, time, and labour costs^4^.

Whereas app-based interventions have important advantages, including low cost, convenience, wide accessibility, and continuous access to the weight-loss program, their effectiveness is currently unclear. Some previous reviews have concluded that app-based interventions are comparable to face-to-face interventions^5^ while others have found that clear conclusions cannot yet be drawn due to the large variability in apps’ content or features^6,7^. One possible reason for this variability is the heterogeneity of the apps or interventions. The features may include ones that have been shown to predict weight loss in traditional behavioural interventions. For instance, one meta-analysis found that more weight was lost in behavioural programs that targeted diet (rather than exercise), included partial or full meal replacement, took place in home settings, and included a dietitian or psychologist^8^. Other factors that predict successful weight loss could be unique to apps—for instance, access to automatically generated progress reports. Identifying the features that predict weight loss could guide the design of future weight-loss apps.

Alternatively, the variability in previous studies could be explained by trials’ different durations. Weight-loss interventions’ effectiveness can be characterised with a time curve or trajectory showing participants’ average weight compared to baseline at different follow-ups. In traditional behavioural weight-loss trials, peak weight loss is typically achieved by 6 months, but weight loss is not always successfully maintained—for instance, around 25% of the lost weight is typically regained by one-year follow-up^9^. Yet, given the high healthcare costs associated with obesity and the effort involved in weight loss, long-term weight loss is of particular interest. To clarify the effectiveness of weight loss apps, reviews should (a) include longer follow-ups and (b) distinguish different time points (follow-ups).

A third possible source of the variability is bias. Typically, meta-analyses assess bias in the included studies, such as bias arising from missing data or the specific methods of outcome measurement (e.g., 7). However, biases arising at the study aggregation level, such as publication bias, are often inadequately addressed, as outlined by systematic reviews of weight loss meta-analyses^5,6^. Recently, a guide for choosing the most appropriate bias correction methods was published along with a bias simulation tool^10^, but this approach has not yet been implemented in the context of weight loss interventions.

To summarise, there is considerable interest in apps that facilitate weight loss, particularly apps that are also effective in the longer term. The present study aimed to estimate the effectiveness of app-based weight-loss interventions in adults and compare their effectiveness to traditional behavioural interventions and no interventions. We included studies that reported follow-ups of at least six months from the start of the intervention, analysing six-month follow-ups (which represent the maximum expected weight loss^9^ separately from longer follow-ups (which represent weight loss sustained in the longer term). We focused on interventions targeting eating behaviours as these have been shown to be more effective than other behavioural interventions^8^. To understand the variability in effects and to identify the features of effective apps, we tested several sample characteristics and app features as moderators of weight loss. We applied three-level meta-analysis, which, unlike more traditional random-effects meta-analysis, does not discard any information in multi-arm studies, retaining the full sample size and minimizing the risk of bias arising due to study selection. We further assessed publication bias and carefully corrected it using methods recommended by recent research^10^.

## Method

### Literature search and study selection

We examined randomised controlled trials (RCTs) of weight loss using smartphone apps. On July 25, 2022, we searched the following databases: Web of Science (Web of Science Core Collection, Essential Science Indicators, and Journal Citation Reports), MEDLINE, PsycArticles, and PsycInfo, the latter three of which we accessed via the EBSCOhost search platform. We searched for articles mentioning (1) a weight outcome (either body weight or BMI), (2) the use of a smartphone app in the title, and (3) either “intervention” or “RCT” in the title or abstract (for EBSCOhost) or topic (for Web of Science). As we were interested in a period when smartphones and smartphone apps had become widely available, the publication period was limited to 2008 onward. The exact algorithms used to search Web of Science and the EBSCOhost databases are listed in supporting information.

Two authors (KA and NI) independently screened the resulting records. We included studies that (a) included a weight-loss intervention delivered at least in part via a smartphone app, (b) randomised participants between intervention and control groups, (c) included either weight or BMI change as an outcome, (d) targeted eating behaviours, (e) had a follow-up assessment at six months or longer after initiation, and (f) were conducted on adults (minimum age 18 years). Studies were excluded if the participants were adolescents, pregnant women or women in the postpartum period, if they assessed weight loss following bariatric surgery, or if they aimed to maintain current weight or prevent weight gain instead of facilitating weight loss. Differences between the two screeners were resolved by discussion; persisting differences were resolved through discussion with a senior researcher (UV). We additionally scanned the reference lists of various earlier reviews found when preparing the study or during the database search.

### Data extraction

Data were extracted independently by KA and NI. For each study, we extracted background information (first author, year of publication, country, study design), information on the interventions (name of the smartphone app or intervention, main content of the intervention) and samples (target population, sample size, participant characteristics), and weight change (weight at initiation and follow-up(s)). Where information was missing or reporting errors were suspected, original study authors were contacted for clarification. To approximate the interventions’ real-world effects and reduce bias by preserving random assignment of participants, we extracted the results of intention-to-treat analyses; studies that only reported the results of per-protocol analyses were excluded. For descriptive statistics and results, means and SDs were extracted; where SDs were missing, they were calculated from CIs or SEs. To assess potential moderating effects, we additionally coded various features of the apps or studies: (1) involvement of medical workers, such as nurses, dietitians, or psychologists in the intervention (yes/no); (2) automatic feedback or progress reports generated by the app (yes/no); (3) calorie counting (none versus rough estimation, e.g. visually, versus precise calorie tracking); (4) prescribed menus or recipes (yes/no); (5) provision of additional weight-loss information via articles (yes/no) or (6) either podcasts or videos (yes/no); (7) social support (none versus ad-hoc groups formed in-app versus inviting friends or family to participate in the trial); and (8) enrollment type (random—all people with excess weight were eligible, versus targeted—people were enrolled based on a specific characteristic like a health condition). Differences between the coders were resolved through discussion.

### Risk of bias assessment

Following the Preferred Reporting Items for Systematic Review and Meta-Analysis (PRISMA) 2020 statement checklist^11^, we assessed the risk of bias in the included studies. Specifically, risk of bias was assessed by NI under the mentorship of TT using the revised Cochrane risk-of-bias tool for randomised trials (RoB 2^12^, covering five domains: (a) bias arising from the randomisation process, (b) bias due to deviations from the intended interventions (effect of assignment to intervention), (c) bias due to missing outcome data, (d) bias in outcome measurement, and (e) bias in the selection of the reported result. The highest risk of bias found in the five domains determines the final score. The RoB 2 was chosen as it is commonly recommended for evaluating bias in RCTs and is suitable for individually randomised, parallel-group, and cluster-randomised trials^12^. Bias was examined in studies reporting weight loss at six months. No automation tools were used besides Google search and the basic keyword search functionality of the webpage. As RoB 2 is based on between-group comparisons (i.e., intervention versus control groups), multiple comparisons were carried out for multi-arm studies.

### Statistical analysis

We first compared app-based to non-app-based interventions and waitilists. Mean difference in weight loss in kg was used for the effect size. For studies reporting the results of more than one app-based intervention, we chose the one that was as app-centric as possible—i.e., had as few components not included in the app (e.g. additional offline support groups) as possible—and compared it to the non-app intervention.

Second, we estimated absolute weight loss in app-based interventions, using raw mean change in weight in kg as the effect size. This enabled a larger sample size than the former analysis as it included all app-based interventions regardless of what type of control intervention was used.

Based on current recommendations for meta-analyses^10^, data were analysed with three-level meta-analytic models. Unlike the traditional random-effects meta-analysis, this approach enables accounting for the dependencies (or non-independent estimates) in multi-arm studies, forgoing the need to randomly select study arms for inclusion which reduces sample size and may introduce bias^13,14^. Three-level models incorporate three sources of variance in effect sizes: sampling variance (level 1), within-study variance or variance in effect sizes reported in the same study (level 2), and between-study variance (level 3). The Knapp and Hartung adjustment was applied to reduce the likelihood of false positives^13^. Within- and between-study heterogeneity in effect sizes was tested with one-sided log-likelihood tests. The distribution of variability in effect sizes between the levels was examined with *I*^2^^15^.

Publication bias was assessed visually with funnel plots and formally tested by adding study precision (sampling variance) as a moderator in the three-level meta-analytic model^14^. As there are currently no agreed-upon guidelines for correcting publication bias in three-level meta-analysis, we conducted random-effects meta-analyses, choosing one effect size at random per study, where bias was found and applied various bias-correction methods to obtain more credible estimates. Publication bias was tested again using Egger’s test. The best-performing bias-correction methods (i.e., ones that minimize the chance of being underpowered or producing false positives^10^ were chosen using an online simulation tool (http://www.shinyapps.org/apps/metaExplorer/) assuming moderate publication bias, high heterogeneity, a small effect size, and medium use of questionable research practices, as suggested by the observed quality of the studies (see the Study quality section).

As a sensitivity analysis, we reran the three-level meta-analytic models excluding outliers and influential cases. Outliers were detected by screening for standardised effect sizes smaller than − 3.29 or larger than 3.29^13^ and by scanning for effects whose confidence intervals did not overlap with those of the pooled estimate. Influential cases were identified with leave-one-out analysis.

We tested potential moderators using three-level metaregression models. Specifically, we tested the effects of interventions’ and apps’ features (described above), as well as study characteristics (gender composition, mean age, mean BMI, trial duration, retention rate, and methodological quality).

Analyses were conducted with R (version 4.2.2) using RStudio. The *metafor* package (version 3.8.1^15^ was used for meta-analyses. The study protocol was not registered. In reporting the study, we follow the guidelines of the PRISMA Statement 2020^11^.

## Results

### Study selection

**Fig. 1:**
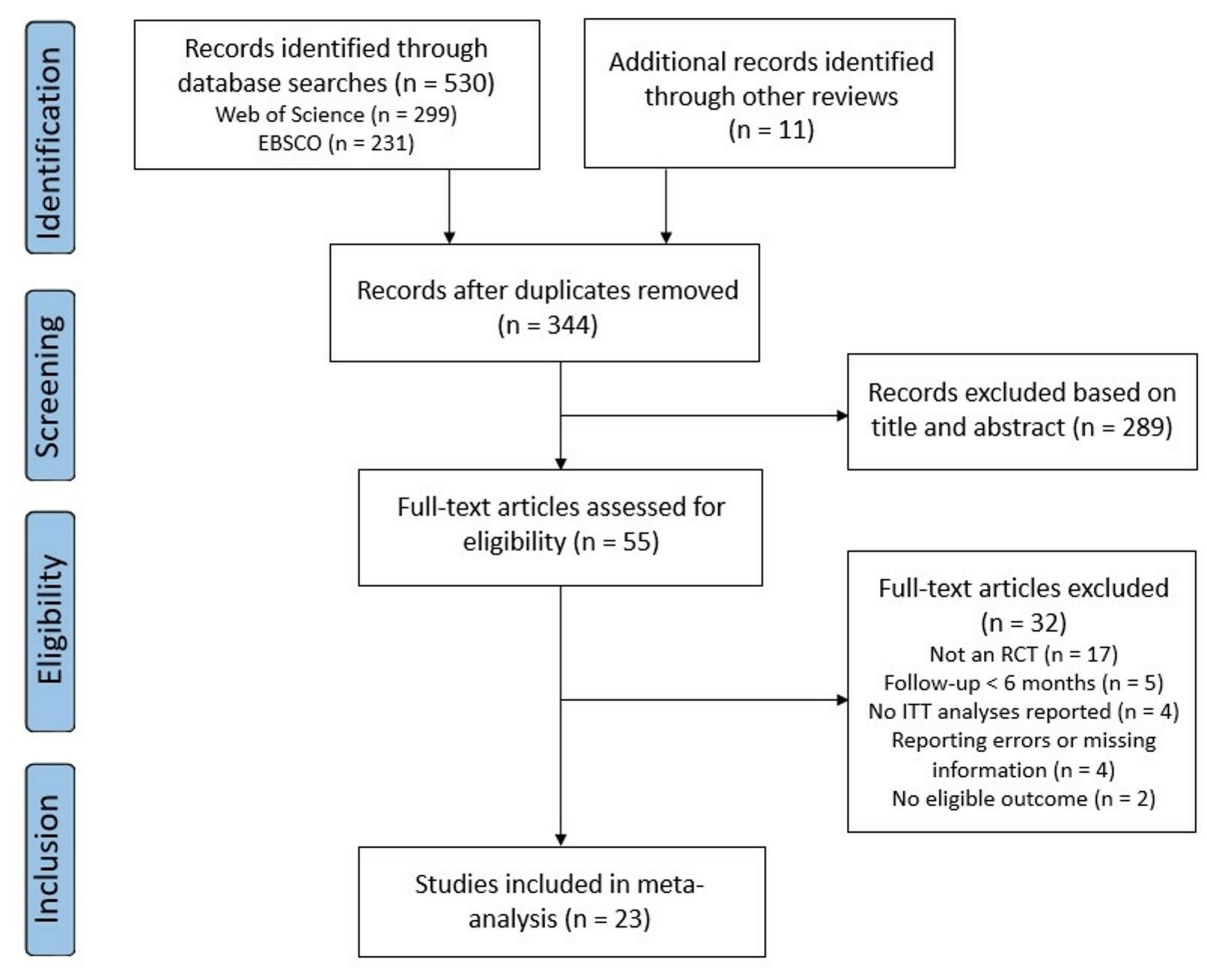
Flowchart of the article selection process. Additional records were identified through previous reviews^16–25^.

Our database searches identified 530 records. 11 additional sources were identified through previous reviews and published meta-analyses. Of the total 541 records, 344 were unique. After excluding records based on titles and abstracts, 55 articles remained and were assessed for eligibility. Twenty-three met our criteria and were included in the meta-analysis. The search and inclusion process is summarised in the flowchart in Fig. 1.

### Sample characteristics

The 23 identified studies contained 30 separate app-based trials. 14 studies reported weight loss at 6 months only, 2 reported weight loss at a longer follow-up time only, and 7 reported weight loss at 6 months as well as a longer follow-up time with the longest follow-up being at 18 months. Total sample sizes were *N* = 2 149 and *N* = 1 697 for the 6-month and longer follow-ups, respectively. The median number of participants was 43 (range 13–298) for the trials with 6-month follow-ups and 114 (range 32–318) for the trials with longer follow-ups. At baseline, the median ages were 49.20 and 55.00 years, median weights 92.50 and 93.65 kg, and median BMIs 33.70 and 33.90 kg/m^2^, respectively, for the trials with 6-month and longer follow-ups. Four studies targeted diet only; 23 additionally targeted other aspects of lifestyle (e.g., physical activity). The samples’ characteristics are summarised in Table 1.

**Table 1:** Main characteristics of included studies.

First author, year	App or study name	Longest follow-up	Country	Population	App-based arm(s)	Non-app arm(s)	*N*	% female	Mean age
Baer, 2020	BMIQ (Intellihealth Inc)	12 months	USA	Primary care patients with overweight or obesity and hypertension or type II diabetes	1) Educational text and video materials, meal plans, sample menus, intake and weight tracking 2) Same plus population health manager support	Information about diet and weight management	840	60.00	59.3
Bennett, 2019	Track	12 months	USA	Adults aged 21–65 years with both obesity and elevated cardiovascular disease risk	Self-monitoring, goals, feedback, smart scale, and counseling calls	Standard care ^1^	351	68.09	50.7
Block, 2015	Alive-PD	6 months	USA	Adults with prediabetes not taking diabetes medications	Emails, tracking, coaching, social support, team competition, health information, and automated phone calls	Waitlist with standard care	339	31.27	46.8
Burke, 2022	SMARTER	12 months	USA	Smartphone users with overweight or obesity (BMI 27–43)	Self-monitoring with feedback	Self-monitoring only	502	79.48	45.0
Carter, 2013	My Meal Mate	6 months	UK	Volunteers with overweight	Goal setting, self-monitoring, and feedback via weekly text messages	Same as app but delivered via 1) website 2) paper diary	128	77.34	41.9
Cho, 2020	Noom	6 months	Korea	Adults at high risk for metabolic syndrome but without diagnosis or pharmacological treatment	Baseline education with 1) diet and exercise self-logging app 2) same app with personalized coaching	Baseline education	129	51.16	49.2
Dunn, 2019	FatSecret; MealLogger	6 months	USA	Adults with overweight or obesity (BMI 25–49.99)	Twice-weekly weight-loss podcasts with diet tracking in 1) calorie-tracking app 2) photo app	NA	43	90.70	42.4
Eisenhauer, 2021	Lose-It!	6 months	USA	Rural men aged 40–69 with overweight or obesity	1) Self-monitoring 2) Self-monitoring with text messaging and discussion group plus and Wi-Fi scale	NA	80	0.00	54.2
Falkenhain, 2021	Weight Watchers; Keyto	24 weeks	USA	Adults with overweight or obesity	An app including recipes, food search, and social support, promoting either 1) a calorie-restricted, low-fat diet 2) a ketogenic Mediterranean diet paired with breath acetone biofeedback	NA	155	70.97	41.0
Ferrante, 2020	SparkPeople	6 months	USA	African American breast cancer survivors with BMI at least 25	Online training session, app for self-monitoring, weekly reminders via text message, activity tracker	Waitlist active control group with weight loss goal and activity tracker	35	100.00	61.5
Hutchesson, 2018	Be Positive Be Healthe (BPBH)	6 months	Australia	Women aged 18–35 (BMI 25.0–34.9)	Goal-setting for weight and behaviour change, quizzes, self-monitoring, individualized feedback for goals and food intake, social support network	Waitlist	57	100.00	27.1
Lim, 2020	Nutritionist Buddy	6 months	Singapore	Adults with nonalcoholic fatty liver disease (NAFLD) and BMI ≥ 23	Diet tracking, videos, daily tips, and nutritionist support in addition to standard care	Standard care	108	37.04	46.5
Lim, 2021	Nutritionist Buddy Diabetes	6 months	Singapore	Adults with type 2 diabetes and BMI ≥ 23	Advisory session, food database, food alternative generator, videos, dietitian support, diabetes management strategies plus digital scale	Introductory advisory session and standard diabetes care plus digital scale	204	35.29	51.2
Lugones-Sanchez, 2022	EVIDENT 3	12 months	Spain	Sedentary adults with overweight or obesity	Food intake monitoring, calorie tracking and feedback, food recommendations, and positive reinforcement, plus activity tracker and brief counseling	Brief counseling group	650	68.46	48.3
Oh, 2015	SmartCare	24 weeks	Republic of Korea	Hospital patients with obesity and metabolic syndrome	Metabolic syndrome treatment, activity and body composition tracking, remote monitoring by professionals, interpretations and recommendations based on the tracked values, monthly and weekly health reports, plus telephone consultations	Metabolic syndrome treatment, activity and body composition tracking	422	49.05	48.6
Ross, 2016	Fitbit	6 months	USA	Adults with overweight or obesity (BMI 27–40)	1) Calorie tracking plus activity monitor and smart scale 2) Same plus phone calls from interventionist	Self-monitoring booklets, pedometer, and scale	80	86.25	51.1
Simpson, 2020	HelpMeDoIt!	12 months	UK	Adults with BMI ≥ 30	Information, goal-setting, monitoring, and social support advice	Healthy lifestyle leaflet	109	69.72	47.3
Spring, 2013	PDA	12 months	USA	Adults with overweight or obesity (BMI 25–40)	Self-monitoring plus biweekly coaching calls	Biweekly sessions	69	14.49	57.7
Spring, 2017	ENGAGED	12 months	USA	Adults with obesity (BMI 30–40)	Self-monitoring dietary intake and body weight, weekly personalised messages, group sessions, calls with trained coaches plus accelerometer	Calorie counting book and self-monitoring diaries with 1) weekly group sessions and calls with trained coaches 2) weight loss target	96	84.38	39.3
Thomas, 2017	Weight Watchers Online	12 months	USA	Adults with overweight or obesity (BMI 27–40)	1) Food intake and body weight tracking 2) Same plus accelerometer providing goals and encouragement	Online educational newsletters	271	77.50	55.0
Thomas, 2019	MyFitnessPal	18 months	USA	Adults with overweight or obesity (BMI 25–45)	Online lessons, self-monitoring, and feedback, plus monthly weigh-ins	Group sessions, self-monitoring	276	82.97	55.1
Turner-McGrievy, 2017	DIET mobile	6 months	USA	Adults with overweight or obesity	Self-monitoring, calorie goal, and behavioral weight loss information via twice-weekly podcasts	Self-monitoring via wearable Bite Counter device, bites-per-day goal, and behavioral weight loss information via twice-weekly podcasts	81	82.72	48.1
Vaz, 2021	Fitbit	6 months	USA	Adults with sedentary jobs (BMI 25–42)	Food photography logs, coaching, and peer support from app, plus outpatient visits, activity trackers, and smartscales	Waitlist with outpatient visits	28	85.71	43.3

The *App-based arm(s)* column briefly describes all app-based trials in the study; the *Non-app arm(s)* column briefly describes all non-app-based trials in the study. For multi-arm studies, different app- or non-app groups are enumerated. All extraced data, including app features tested as moderators and more detailed descriptions of the interventions, are available at https://osf.io/amg54/.

^1^Standard care – weight-loss advice focusing on diet and other lifestyle aspects.

### Comparing app-based interventions to waitlist and non-app-based interventions

Significantly more weight was lost in app-based interventions than on waitlists (mean difference 2.07 kg, 95% CI 1.09–3.05, *t*(11) = 4.64, *p* <.001, *k* = 12) and in non-app-based interventions (1.64 kg, 95% CI 0.25–3.03, *t*(14) = 2.53, *p* =.024, *k* = 15) at the 6-month follow-up. The Egger test showed no publication bias in either analysis (intercept’s *b* = 3.94, 95% CI −17.47–9.59, *t* = −0.57, *p* =.581 for comparison with waitlist and *b* = 0.07, 95% CI −0.84–0.71, *t* = −0.17, *p* =.869 for comparison with non-app-based interventions). The funnel plots for the comparison meta-analyses are shown in Figures S1–S2. We did not conduct meta-analyses for longer follow-ups due to the low number of studies with available data.

### Weight loss in app-based interventions

#### Six-month follow-up

**Fig. 2:**
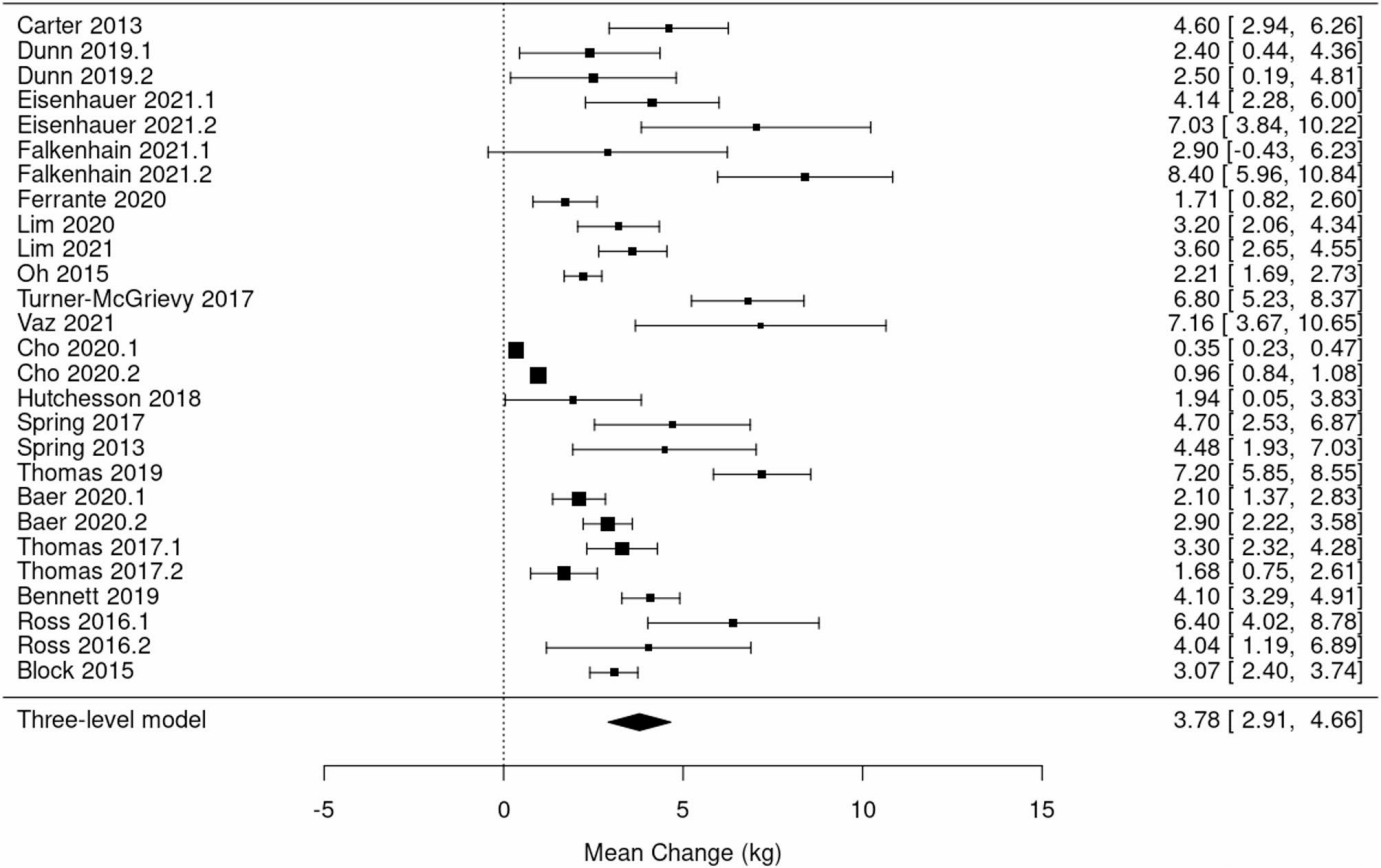
Forest plot of three-level meta-analysis (6-month follow-up).

**Fig. 3:**
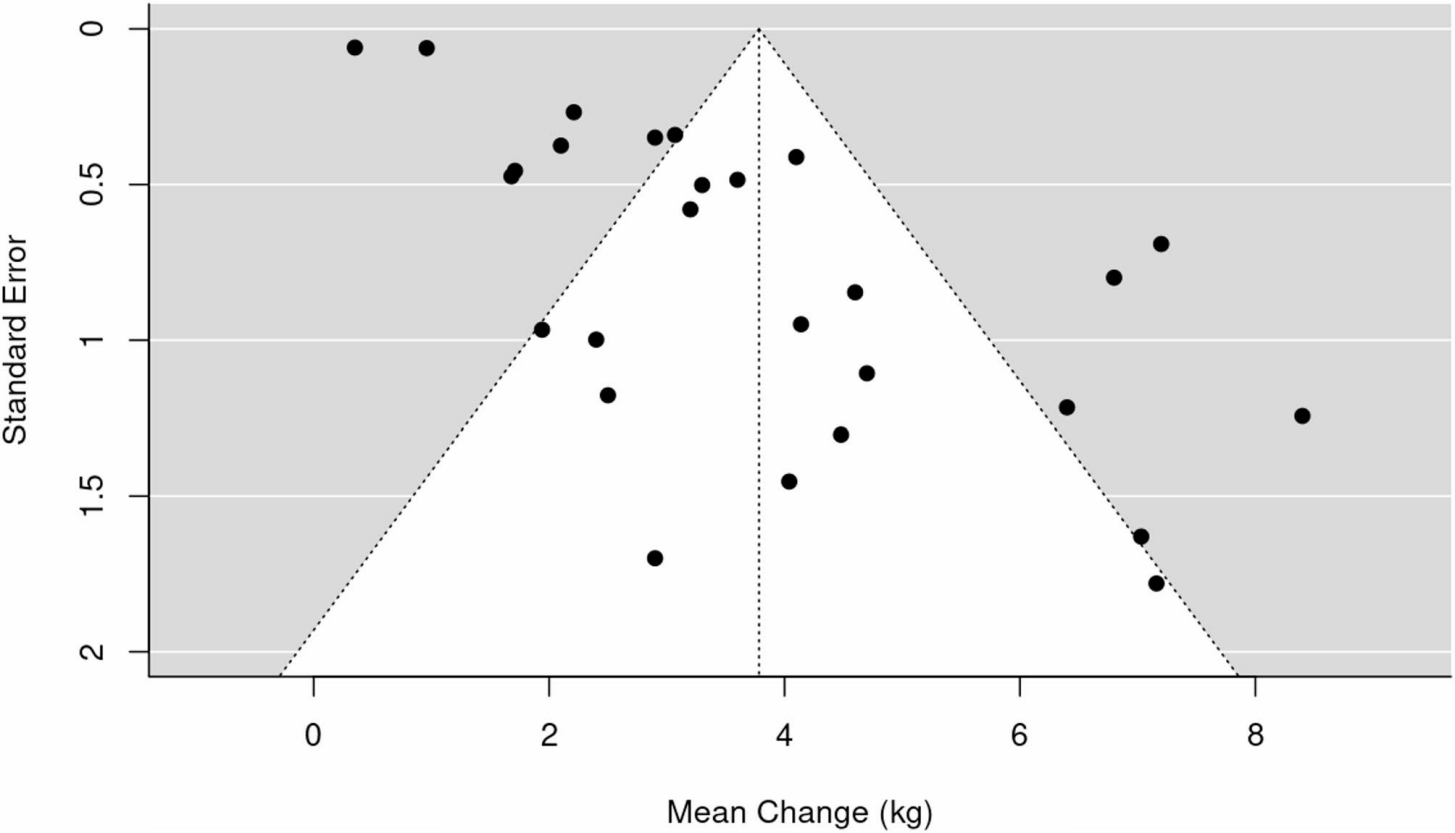
Funnel plot for weight loss in app-based interventions (6-month follow-up).

The meta-analytic models and their bias-correction tests are summarised in Table 2. The three-level model (*k* = 27) indicated mean weight loss of 3.78 kg (95% CI 2.91–4.66 kg, *t*(26) = 8.90, *p* <.001 from baseline; Fig. 2). However, publication bias assessment suggested this effect was inflated as the funnel plot was highly asymmetric (Fig. 3) and study precision had a significant moderating effect (*F*(1,25) = 8.00, *p* =.001). Effect sizes were highly heterogeneous (*I*^*2*^ = 97.89%) with most (78.31%) of the variability being at the between-study level (σ^2^_level2_ = 2.57, *p* <.001), some at the within-study level (19.59%; σ^2^_level3_ = 0.63, *p* =.037), and little being accounted for by sampling variance (2.11%).

**Table 2:** Meta-analysis of weight loss in app-based interventions.

Model	Test’s best performance	k	I^2^	b	95% CI		t- or z-statistic	*p*	Test of bias (*p*)
					Lower limit	Upper limit			
6-month follow-up									
Three-level	–	27	97.89	3.78	2.91	4.66	8.90	< 0.001	0.009
Three-level (excl. 9 trials)	–	18	38.22	3.60	3.12	4.08	15.84	< 0.001	0.026
Random effects	H1	20	95.88	3.87	2.94	4.80	8.15	< 0.001	< 0.001
Trim-and-fill	H1	20	95.88	3.87	2.94	4.80	8.15	< 0.001	–
3PSM	H0	20	–	2.57	0.43	4.72	2.35	0.019	–
PET	H0	20	–	0.63	0.20	1.06	2.85	0.011	–
PET-PEESE	H0	20	89.83	1.47	0.04	2.90	2.01	0.044	–
Longer follow-ups									
Three-level	–	11	87.71	2.64	1.70	3.57	6.3	< 0.001	0.350

Test’s best performance indicates whether the bias-correction method performs best under the null hypothesis (H0) and minimizes false positives or under the alternative hypothesis (H1) and maximizes statistical power. Test of bias entailed adding SE as a predictor to the three-level models or Egger’s test for random-effects models.

No outliers were detected based on standardised weight-loss estimates but eight trials’ CIs did not overlap with the pooled estimate^26–33^. Additionally, while no trials were influential in terms of effect size in leave-one-out analysis (Figure S3), the app-only trial reported by Cho et al.^26^ was influential in terms of heterogeneity (Figure S4). The sensitivity analysis excluding the outliers and influential studies showed a slightly lower effect (mean weight loss 3.60 kg, 95% CI 3.12–4.08 kg, *t*(17) = 15.84, *p* <.001, *k* = 18); because the difference was small, we did not exclude the studies from further analyses. However, total heterogeneity dropped dramatically to *I*^*2*^ = 38.22% with 61.77% of the remaining variability being accounted for by sampling variance and between-study variability being 38.22% (non-significant; σ^2^_level2_ = 0.36, *p* <.001). Thus, heterogeneity was not entirely accounted for by single outlying studies. Publication bias was still present as indicated by the significant moderating effect of precision: *F*(1,16) = 6.05, *p* =.026.

Due to the significant publication bias, we proceeded with a random-effects meta-analysis. However, publication bias was still very strongly present (Egger’s test *b* = 9.61, 95% CI 5.89–13.33 kg, *t* = 5.06, *p* <.001), necessitating further bias correction. Based on the simulation (shown in Figures S5 and S6 in supporting information), we applied the following bias-correction methods. First, three methods—the precision-effect test (PET), the precision-effect test/precision effect estimate with standard error (PET-PEESE), and the three-parameter selection model (3PSM)—were applied that perform best under the null hypothesis (i.e. minimise false positive rate) (Figure S5 and S6, upper panels). These methods suggested that the true effect ranges from 0.63 to 2.57 kg (Table 2). However, while these methods minimise false positives, more accurate estimates may be obtained with methods that maximise statistical power and perform best under the alternative hypothesis^10^. The simulation suggested that the random-effects model itself, as well as trim-and-fill, were most appropriate under current conditions (Figures S5 and S6, lower panels). Both methods estimated the effect to be 3.87 kg (Table 2).

#### Longer follow-ups

**Fig. 4:**
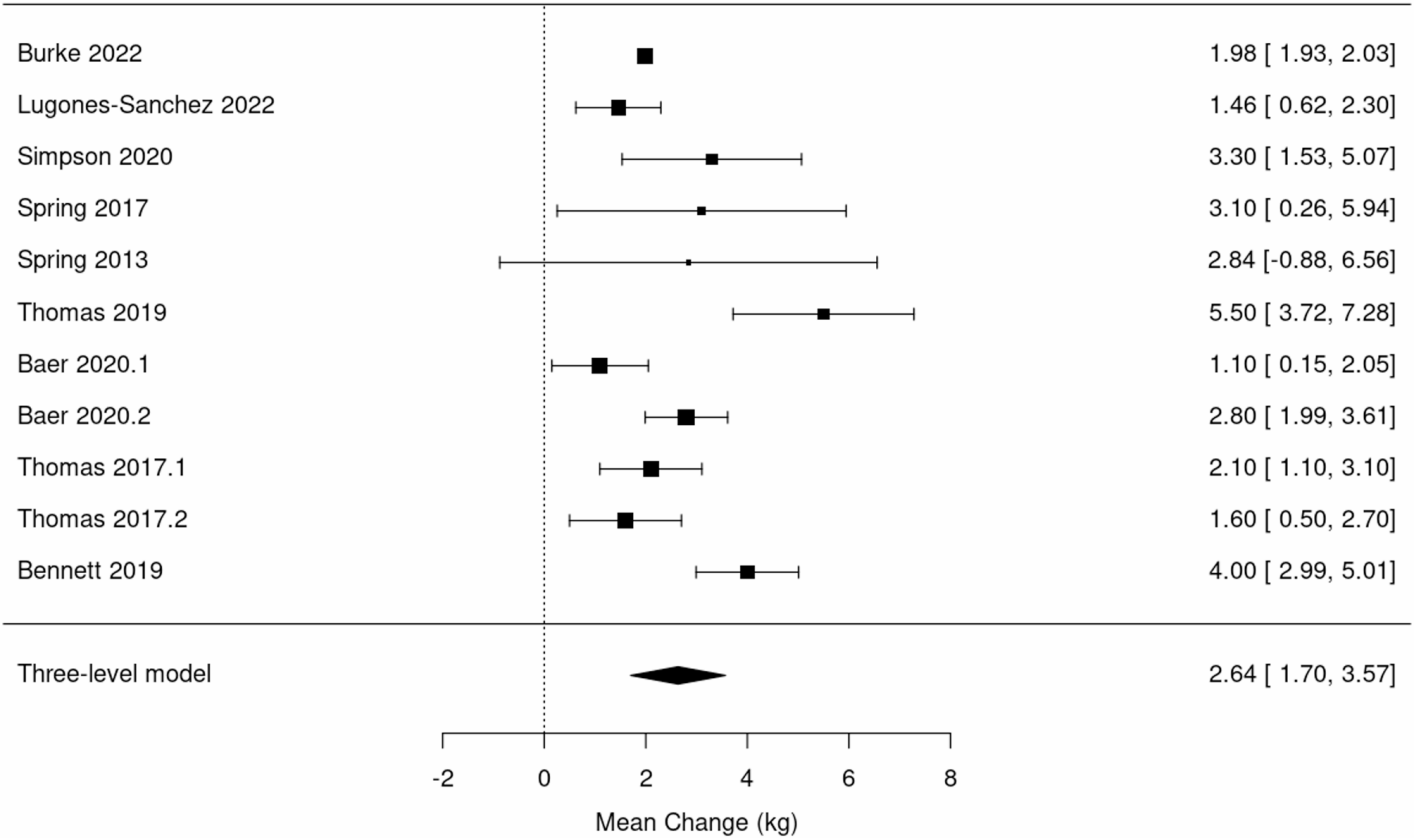
Forest plot of three-level meta-analysis (follow-ups over 6 months).

**Fig. 5:**
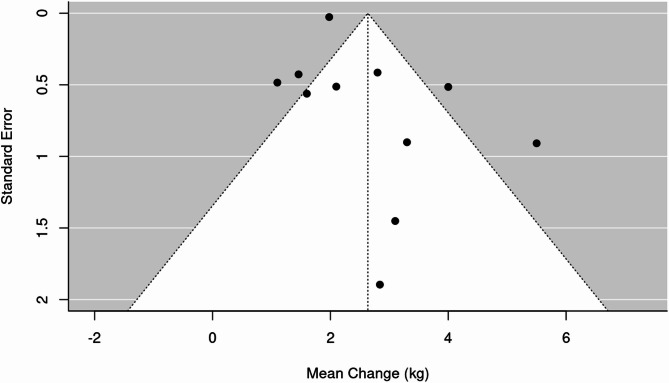
Funnel plot for weight loss in app-based interventions (follow-ups over 6 months).

The three-level model (*k* = 11) yielded an estimate of 2.64 kg for mean weight loss (95% CI 3.19–4.22 kg, *t*(10) = 6.30, *p* <.001) at longer follow-ups (Fig. 4). Total heterogeneity was *I*^*2*^ = 87.71%; there was significant between-study heterogeneity (σ^2^_level3_ = 0.72, *p* =.038; 51.38% of total variability) but not within-study heterogeneity (σ^2^_level2_ = 0.51, *p* =.376; 36.34% of total variability); sampling variance accounted for 12.29% of total heterogeneity. Additional bias correction was unnecessary as there was no evidence for significant publication bias: study precision did not moderate the effect in the three-level model (*F*(1,9) = 0.97, *p* =.350) and the funnel plot (Fig. 5) was roughly symmetrical.

### Study quality

As summarised in Figure S7, the studies were of mixed quality. Six of the 28 comparisons had a low risk of bias, four were rated as having some concerns of bias, and 18 had a high risk of bias. There were concerns about randomisation in one cluster-randomised trial where patients were randomised before their eligibility for inclusion was checked, leading to a possibility of imbalanced groups^32^. Some deviations from the protocol were found^27,34,35^. In some cases, we were unable to confirm study protocols^16,26,29,31,36^ which suggests a possibility of selective reporting.

Most commonly, however, problems were found with participant exclusions and handling missing data. Some studies gave vague or no reasons for loss to follow-up^27,28,31,34,37^ or did not report how missing data were handled when cases were likely missing not at random^28,31,37–39^. One study did not justify the exclusion of participants from intention-to-treat analyses^26^ and one provided conflicting information regarding participant exclusion from analyses^40^. Other studies received a higher risk of bias rating due to inappropriate or unjustified usage of multiple imputations^32,39,40^ or due to carrying forward last or baseline observations^27,35,41^. Problems with unbalanced retention rates between groups were additionally identified^27,28,32,35,37,42^.

Issues with outcome measurement were also common. Although the staff measuring participants’ weights was usually blinded to their group, this was not the case in all studies^28,34,38,40,43^, leaving the possibility of skewed results. In three studies, participants self-reported their weights, whereas there was no mention of any instructions provided to them^34,35,40^. In another three studies, the outcome was measured during an interval of several weeks across participants^32,34,36^.

### Moderation analysis

Finally, we explored each moderator’s effect in a separate three-level model (Table 3 and Figures S8–S21). After applying false discovery rate correction, none of the app features (involvement of medical workers, automatic feedback or progress reports, type of calorie counting, prescribed menus or recipes, weight-loss articles, podcasts or videos, social support) or study or sample characteristics (mean BMI or age at baseline, percent of female participants, trial duration, enrollment type, retention rate at 6 months) had a significant effect on weight loss. Similarly, study quality had no moderating effect, indicating that weight loss did not differ between higher- and lower-quality studies. It is worth noting that the small number of studies may have limited our ability to detect moderation effects.

**Table 3:** Moderation Effects.

Moderator	b	t^1^	*p* ^2^
App features			
Involvement of medical workers	0.65	1.24	0.796
Automatic reports	− 0.29	− 0.35	0.882
Type of calorie counting	0.02	0.03	0.980
Prescribed menus or recipes	0.60	− 0.77	0.805
Weight-loss articles	− 0.81	− 0.67	0.805
Weight-loss podcasts or videos	0.60	0.68	0.805
Social support	0.56	0.84	0.805
Study and sample characteristics			
Baseline mean BMI	0.34	2.57	0.162
Baseline mean age	− 0.01	− 0.23	0.888
% female	0.43	0.27	0.888
Trial duration	0.08	0.66	0.842
Enrollment type	− 1.84	− 2.38	0.162
Retention rate at 6 months	1.27	0.36	0.888
Study quality	0.49	0.89	0.842

The moderator’s effects are illustrated in Figures S8–S21. App features were coded as 0/1 (not used/used) for involvement of medical workers, automatic reports, prescribed menus or recipes, articles, podcasts or videos; calorie counting was coded as 0/1/2 (none/rough estimation/precise tracking); social support was coded as 0/1/2 (none/ad-hoc groups/inviting friends or family). Enrollment type was coded as 0/1 (random/targeted).

^1^*df* = 22 for type of calorie counting and study quality; *df* = 25 otherwise.

^2^False discovery rate correction applied.

## Discussion

This meta-analysis estimated average weight loss in smartphone app-based programs and compared these programs’ effectiveness to traditional (non-app-based) behavioural treatments and waitlists. Results showed that apps were more effective than both alternatives. Different methods correcting for publication bias suggested that the average weight loss ranged from roughly 0.5 to 4 kg at six months; the average weight loss at longer follow-ups was approximately 2.5 kg. Although apps’ typical effects may not be large enough to reduce body weight from the obesity range to the normal weight range, they nevertheless appear useful for facilitating weight loss and could be cost-effective alternatives to standard behavioural treatments. We discuss below when and for whom weight-loss apps may be most beneficial.

App-based interventions resulted in greater weight loss than waitlists (mean difference 2.07 kg) and non-app interventions (1.64 kg) at the six-month follow-up. To compare, a recent review of reviews reported a larger contrast between apps and no treatment (4.32 kg) and a marginal difference from face-to-face interventions^5^. The comparatively smaller difference between apps and waitlists we found likely reflects our focus on intention-to-treat analyses, which capture real-world effects rather than outcomes among the most committed participants. The observed advantage of apps over non-app interventions may stem from the relative simplicity of the latter—for instance, some control treatments in our included studies had fewer components than app-based ones. Thus, their lower effectiveness could reflect their lower intensiveness rather than true differences and should be interpreted cautiously. Discrepancies between the current study and the previous review^5^ may also be explained by the broader scope of the latter or by the different handling of intervention duration: the previous review included 3- to 24-month follow-ups but did not analyse them separately.

Our estimates for apps’ net effects ranged from 0.63 to 3.87 kg at six months and averaged 2.64 kg at longer follow-ups. Although our study included a larger number of technology-based trials than previous ones^7,44^ as it was not restricted by the choice of control treatments, the publication bias we detected at six months complicates drawing precise conclusions. We propose two ways to resolve the wide range of the six-month estimates. First, we could average the different estimates, which yields 2.48 kg, although this may underestimate the true effect as some of the methods prioritized minimising false positives over maximising statistical power^10^. Second, assuming 25% weight regain from six to 12 months^9^, we could calculate the six-month effect from the longer follow-up estimate (which was most often 12 months), which yields 2.64 kg/0.75 = 3.52 kg. Considering these alternatives, weight loss at six months could average between 2.5 and 3.5 kg. Despite the uncertainty, it is reassuring that both estimates fall within the range of the six-month estimates we obtained.

These effects exceed some previous estimates of around 2 kg across varying time intervals^7,44^. Our estimates may be higher due to our focus on eating behaviour, which tends to be a more effective target than its most common alternative—exercise^8^. Still, even the higher estimate suggests that not enough weight is lost with apps to help people with obesity into the normal weight range or eliminate the associated health risks. Such cases may still require surgical or drug treatments^4,8^ which, despite their higher costs and risks, tend to have larger effects.

However, despite their limited average effects, apps may be useful in several situations. First, they may suit people who aim for modest weight loss, including people with obesity who need to lose weight to qualify for bariatric surgery. Second, weight-loss outcomes vary, and some people, such as those with higher self-discipline, may be better able to adhere to the intervention and thus achieve substantially greater results than the average estimates would predict. Third, all other interventions eventually need long-term behavioural support, and apps could serve as a cost-effective weight maintenance tool following these other treatments. It should be noted that a potential limitation of app-based programs is their greater suitability for regular smartphone users than for people with limited digital skills or the ability to use smartphones.

Perhaps the most unexpected result of the current study is the lack of moderation effects despite the inclusion of various study, sample, and intervention characteristics. One possible explanation is that the specific features of app-based interventions do not matter: rather than the specific content of the apps, weight loss may be driven by increased attention to weight loss. In this case, apps could forgo more expensive features such as continuous support from medical workers. However, the fact that we did not find moderation effects that could have been expected suggests that our analyses may have been underpowered to detect them. For example, despite the large variation in the proportion of women included in the trials, we did not detect any moderating effect of gender composition, which could be expected given that men typically lose more weight than women during interventions^45,46^. This possibility is further supported by a meta-analysis of 169 behavioural trials found that six of the 28 tested moderators were related to weight loss, but detecting a moderator effect associated with a 1 kg difference required more than 5 000 participants^8^—over twice the sample size of the current meta-analysis. Indeed, given the limited effects of apps, moderation effects may be small. Thus, future analyses should explore potential moderators further. For a more systematic test of moderation, a framework of interventions’ features could be developed; future interventions could map these features, and future meta-analyses could clarify their role.

It is important to consider the scope and limitations of the current research. Our meta-analysis included studies published between 2008 and 2022. The observed pattern—modest average weight loss with substantial heterogeneity—aligns with other reviews^5,44^, suggesting it is unlikely that newer evidence would substantially alter the overall conclusions unless new and more effective weight-loss techniques are adopted. Still, different results may be obtained as the field evolves. For instance, once enough evidence has accumulated, future studies could test whether the incorporation of artificial intelligence (e.g.^47^) may affect the outcomes. Further, our study had a relatively narrow focus as it only included RCTs that targeted eating behaviour, focused on adult populations with overweight or obesity, and had a minimum follow-up of six months. We specifically focused on longer follow-ups due to interest in and limited prior knowledge of longer-term weight loss but studies with longer follow-ups (e.g. 12 months or more) were scarce. Studies with longer follow-ups are needed to clarify if weight loss with apps follows a similar weight loss trajectory as other methods. Ultimately, the trajectories have implications for the treatments’ potential uses: differences may pinpoint whether one approach is more suitable for rapid weight loss or long-term maintenance than another (e.g. when comparing app and non-app behavioural interventions). However, as the current and other related meta-analyses suggest^5,48^, more studies with longer-term follow-ups should be undertaken.

Further, because the three-level approach we employed is unable to address the considerable publication bias, we had to rely on various bias-correction methods that randomly select (and thus discard some) trials in multi-arm studies in the six-month data. However, as random-effects models are the standard approach in meta-analyses, this is a common limitation. The steps taken to detect and correct bias altogether allow reasonable confidence in the findings, and future app-based trials can use our estimates for sample size calculations. Still, future studies should follow strict protocols to ensure high quality and credible results.

Taken together, the results suggest that smartphone apps can facilitate weight loss. How they compare to traditional behavioural interventions in the long term (across several years) is still unclear considering the limited available evidence, but they seem a promising cost-effective alternative. Besides being used on their own to achieve modest weight loss, or potentially more substantial weight loss in people with greater adherence to the programs, smartphone apps could accompany bariatric surgery or drug treatments for weight maintenance. Considering the available evidence and remaining uncertainties, we recommend conducting more long-term studies, adhering to study protocols, and further examining sources of variability in effects to clarify the role of participant and intervention characteristics. Once more evidence is available, apps’ average weight-loss trajectory can be clarified and compared to other interventions, which will ultimately help position apps within the vast landscape of weight-loss treatments.

## Supplementary Information

Below is the link to the electronic supplementary material.

Supplementary Material 1

## Data Availability

Study materials, including the data and code, are available at https://osf.io/amg54.
