# Supplementary material for: Meta-analysis of smartphone applications targeting eating behaviour for weight loss
Source: Sci Rep. 2026 Jun 5;16:25645. doi: 10.1038/s41598-026-56407-7 (PMC13478153; doi:10.1038/s41598-026-56407-7)
Supplement: Supplementary file 1 [file 41598_2026_56407_MOESM1_ESM.pdf]

Supporting Information

Meta-analysis of smartphone applications targeting eating behaviour for weight loss

Kadri Arumäe, Neeme Ilves, Eno-Martin Lotman, Triin Viskus, Tõnu Esko, Priit Palta, Taavi Tillmann, Uku Vainik

Search algorithms

Web of Knowledge

(((((((TI=(weight OR BMI OR "body mass")) AND TI=(app OR application OR mobile OR smartphone OR mhealth)) AND TS=(intervention OR trial OR RCT)) AND PY=(2008-2022)) NOT TI=(review OR meta-analysis)) NOT TI=(child OR pediatric OR infan* OR pregnan*))) NOT TI=(protocol)

EBSCOhost (Medline, PsycArticles, PsycInfo)

Search algorithm: TI (weight OR BMI OR "body mass") AND TI (app OR application OR mobile OR smartphone OR mhealth) AND (TI (intervention OR trial OR RCT) OR AB (intervention OR trial OR RCT)) NOT TI (review OR meta-analysis OR child* OR pediatric* OR infan* OR pregnan* OR gestation* OR postpartum* OR protocol)

Additional limiter: Date of Publication: 20080101-20220731

Figure S1

Funnel Plot for Weight Loss in App-based Interventions Versus Waitlists (6-month Follow-up)

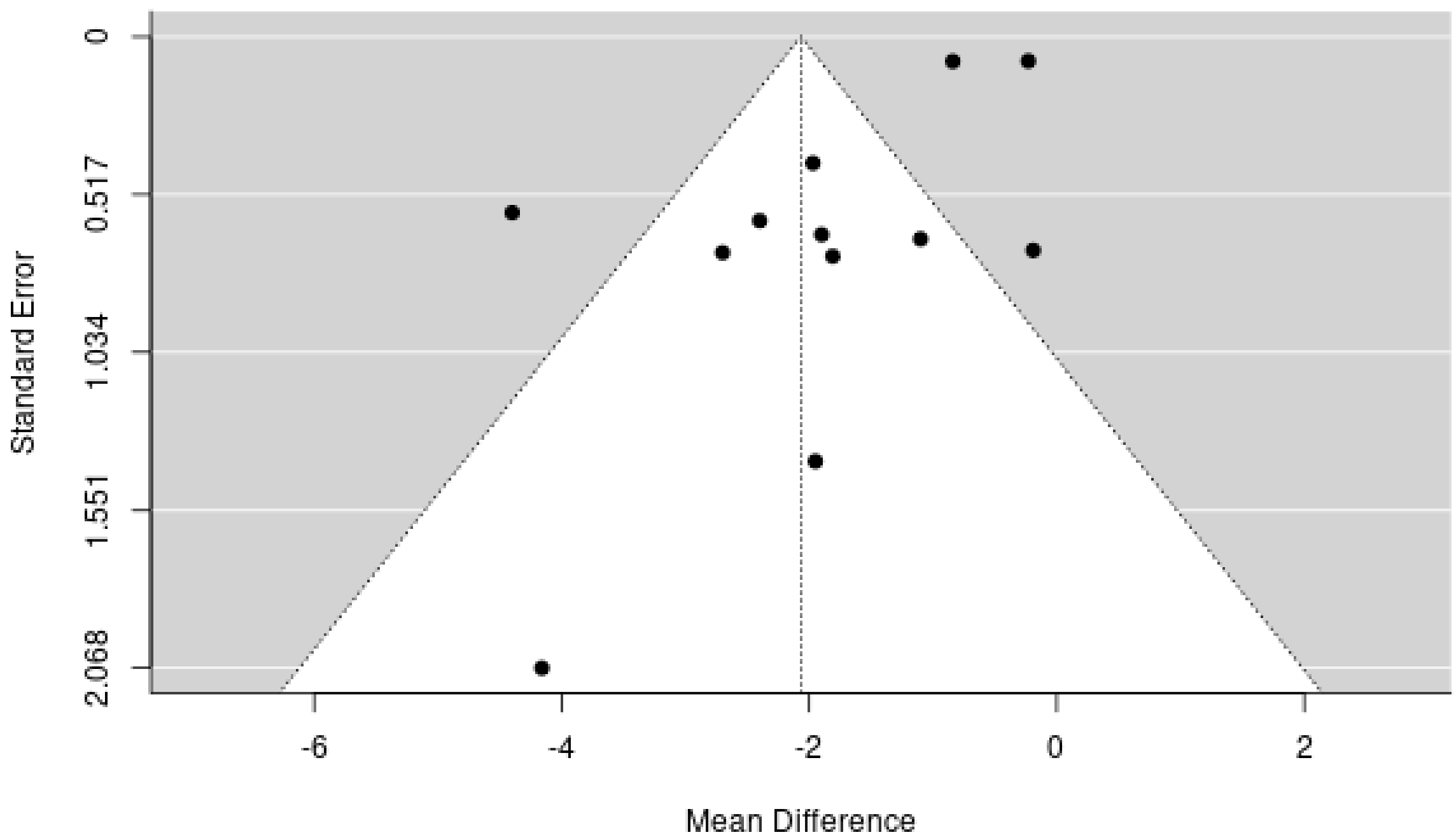

Note. Studies comparing app-based interventions to waitlists are displayed. Mean difference = -
-2.07 kg.

Figure S2

Funnel Plot for Weight Loss in App-based Versus Non-app-based Interventions (6-month Follow-up)

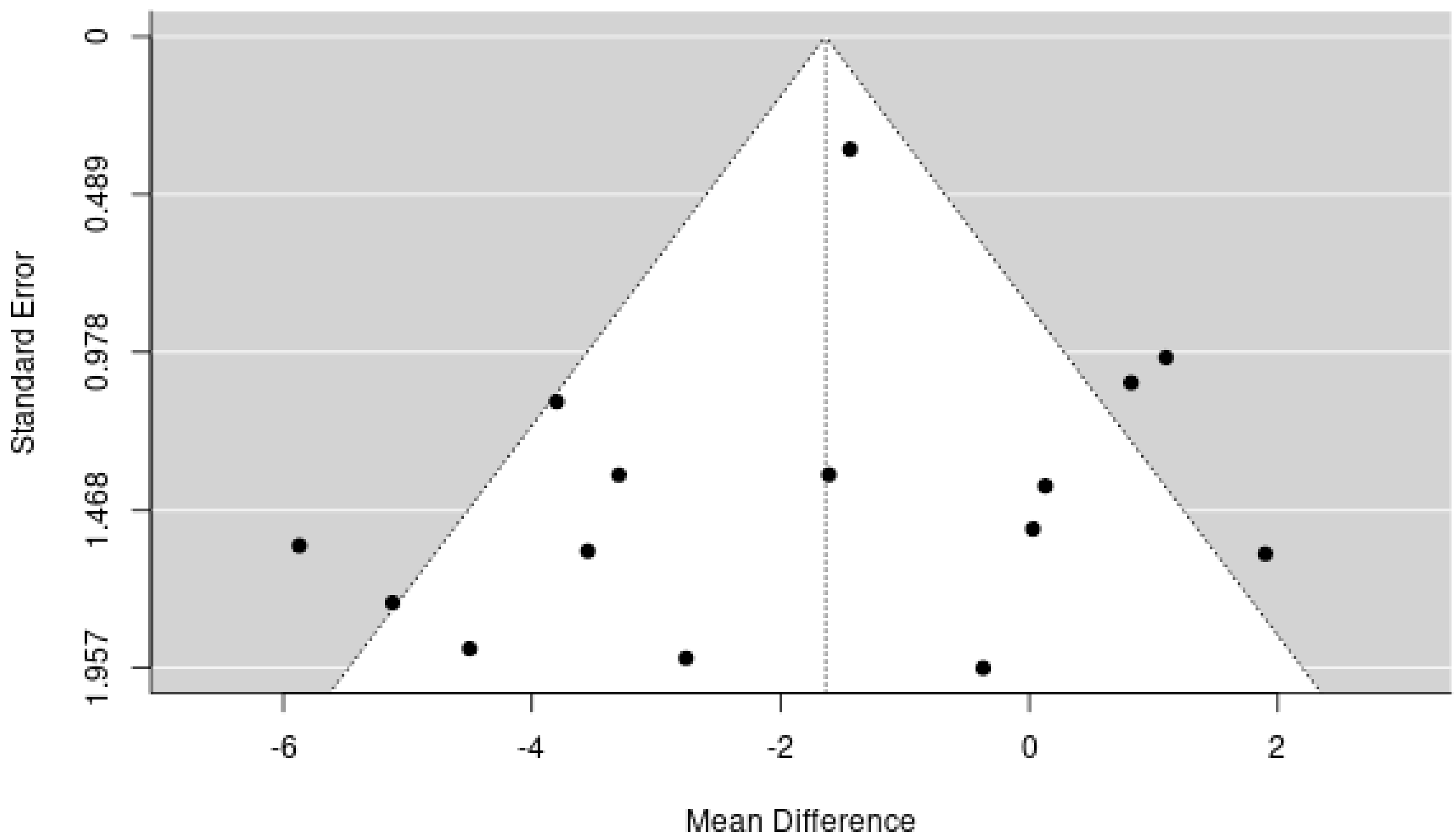

Note. Studies comparing app-based interventions to non-app-based interventions are displayed. Mean difference = -1.64 kg.

Figure S3

Leave-one-out Analysis for Effect Size (6-month Follow-up)

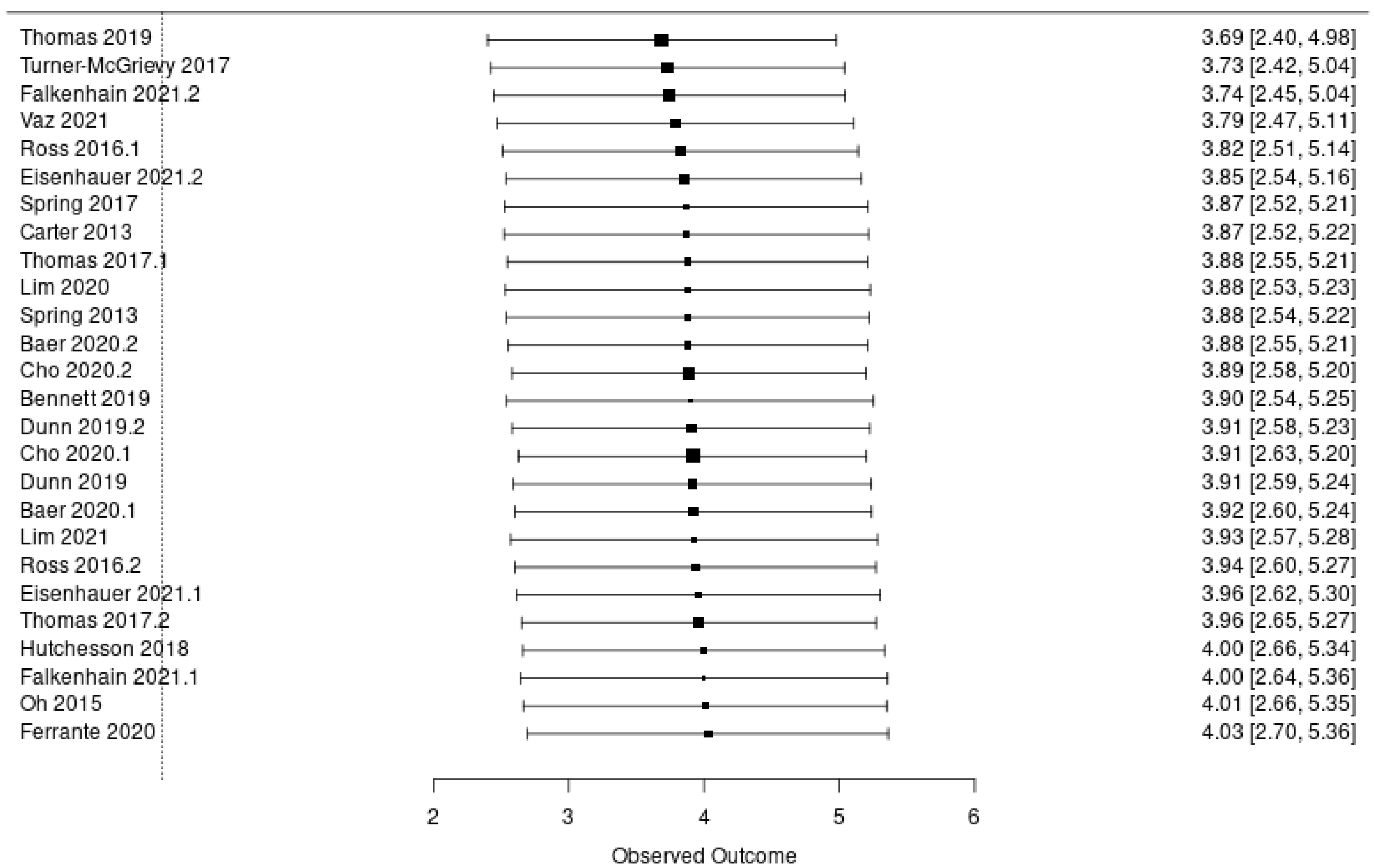

Note. Analysis to identify trials with disproportionate influence on pooled effect size. No trials were found to have disproportionate effects.

Figure S4

Leave-one-out Analysis for Heterogeneity (6-month Follow-up)

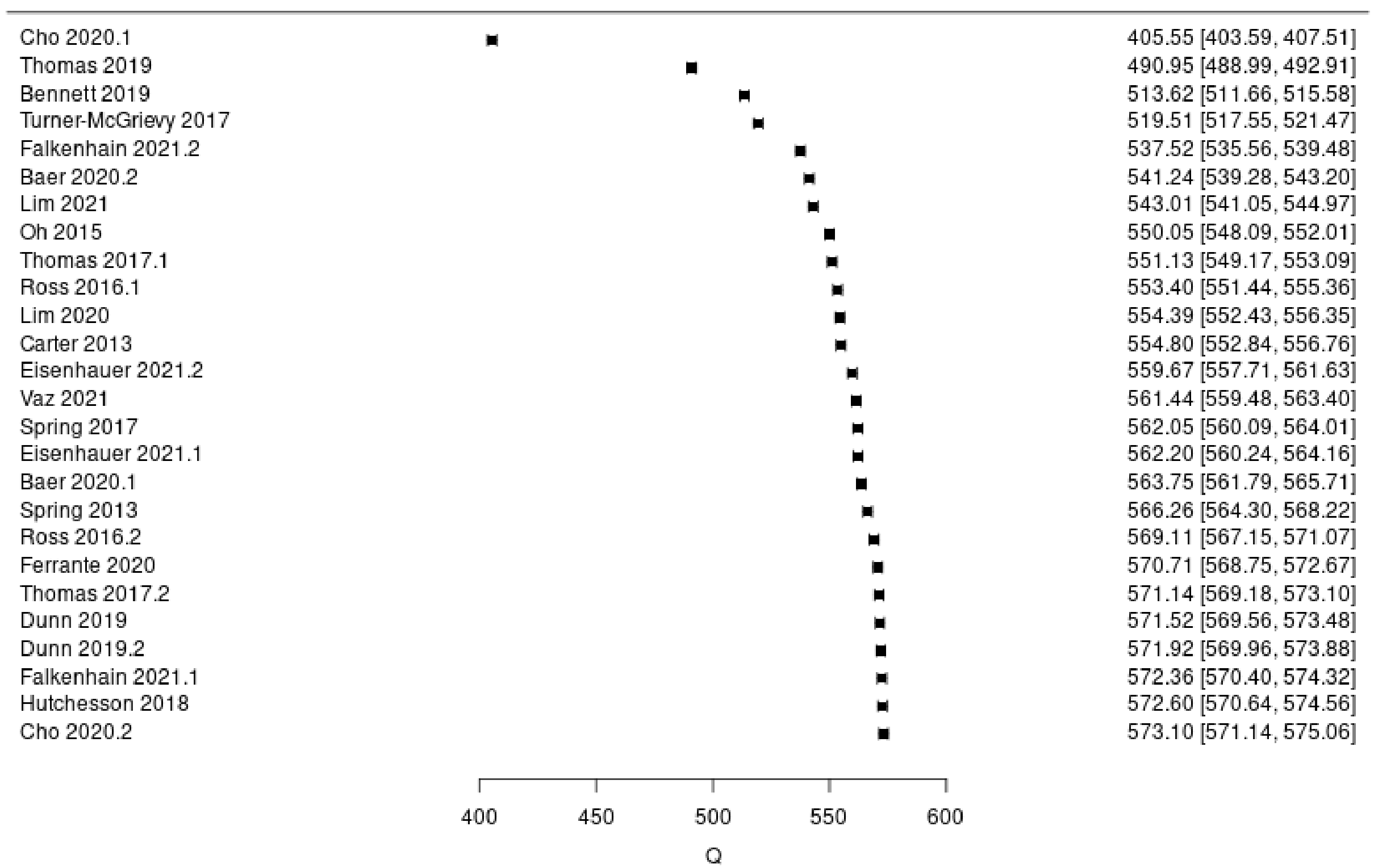

Note. Analysis to identify trials with disproportionate influence on pooled heterogeneity. A trial reported by Cho et al. (shown on the figure as *Cho 2020.1*) was found to have a disproportionate effect.

Figure S5

Simulated Performance of Bias-correction Methods at k *= 30*

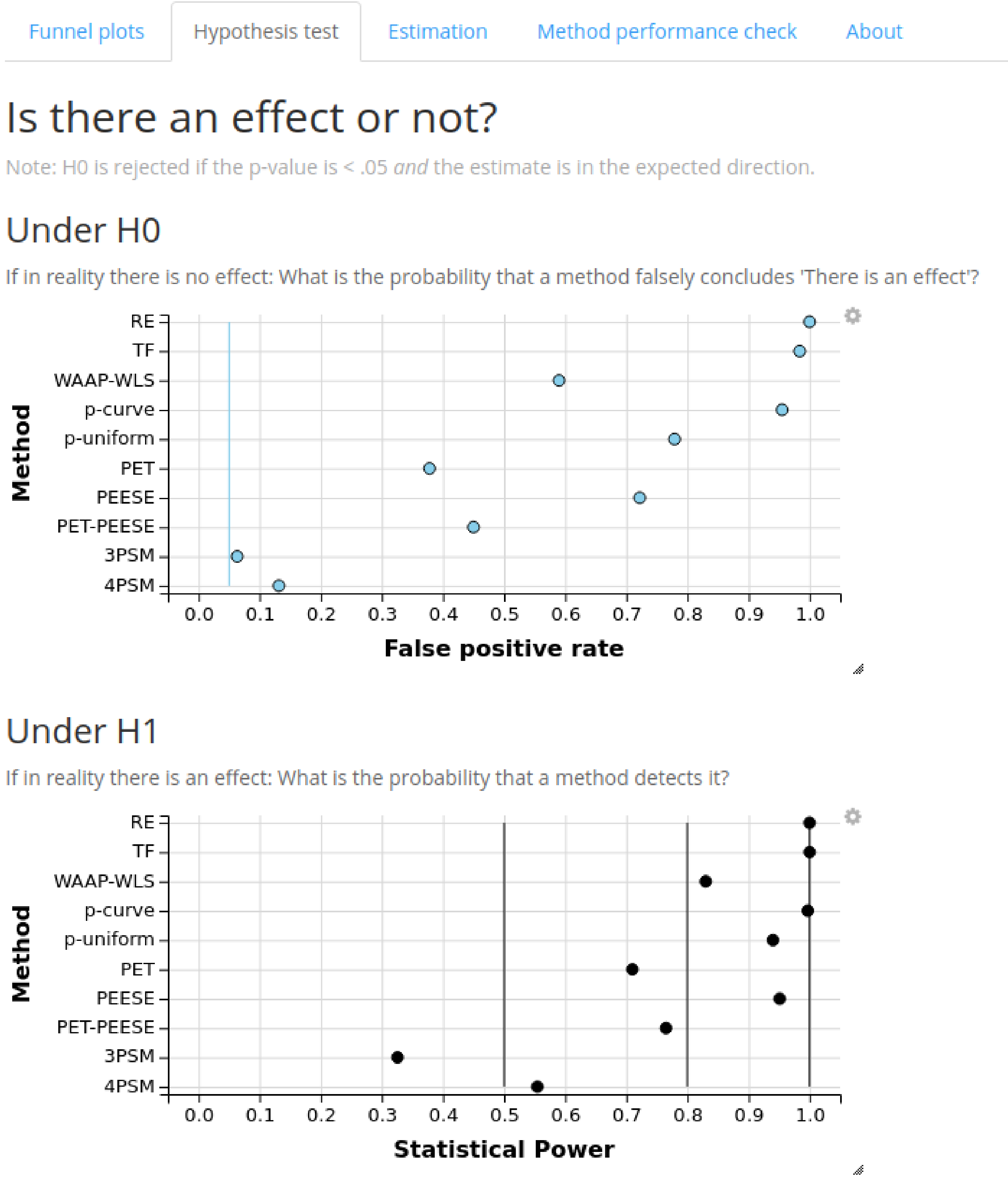

Note. A simulation to guide the selection of bias correction methods given *k* = 30 studies. We assumed moderate publication bias, high heterogeneity, a small effect size, and medium use of questionable research practices. The simulation tool is available at http://www.shinyapps.org/apps/metaExplorer/.

Figure S6

Simulated Performance of Bias-correction Methods at k *= 10*

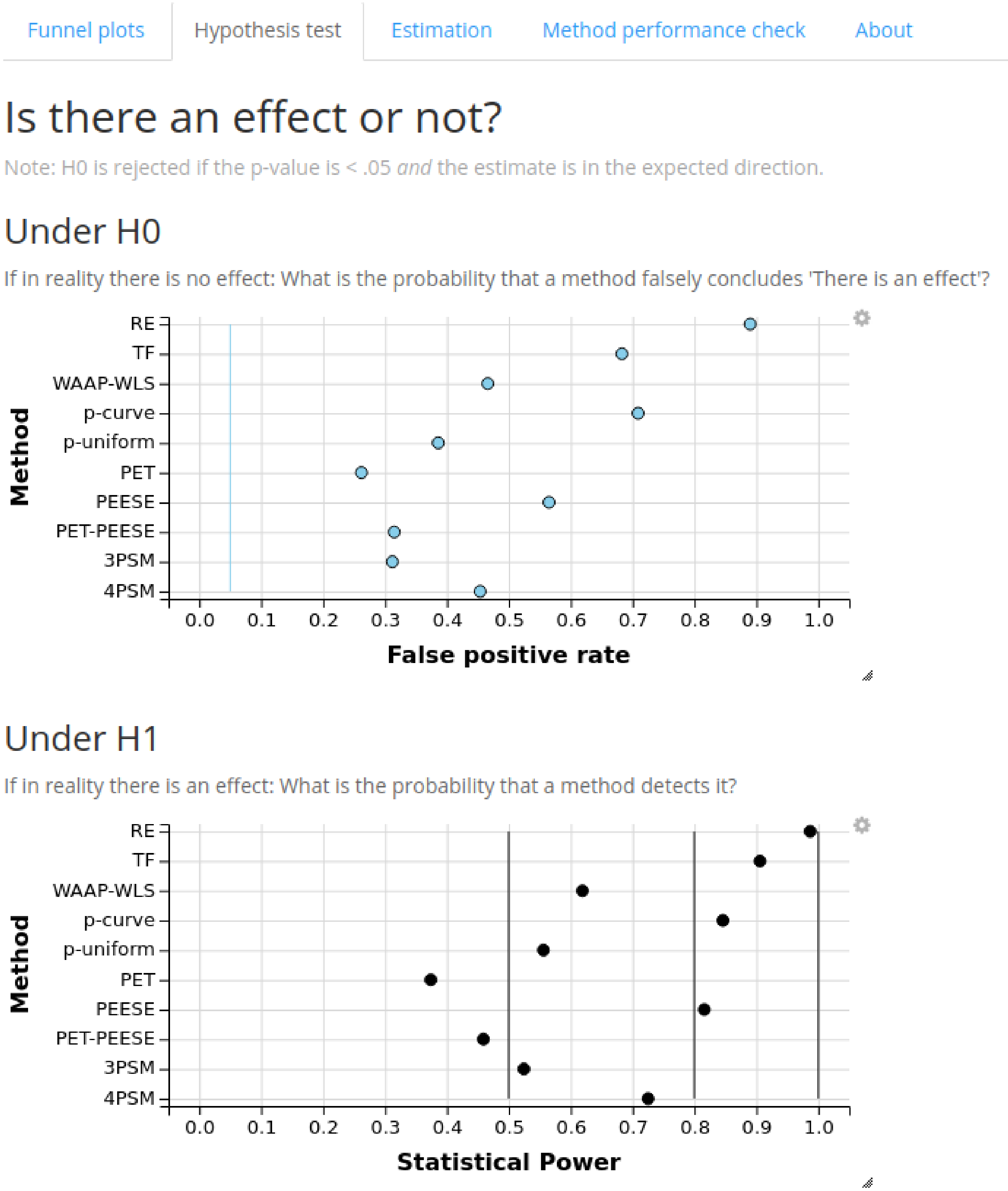

Note. A simulation to guide the selection of bias correction methods given $k = 10$ studies. We assumed moderate publication bias, high heterogeneity, a small effect size, and medium use of questionable research practices. The simulation tool is available at http://www.shinyapps.org/apps/metaExplorer/.

Figure S7

Risk of Bias

First author, year	Intervention	Control treatment	1	2	3	4	5	F
Baer, 2020	Online program only	Usual care	Red	Green	Red	Yellow	Green	Red
Baer, 2020	Combined online program	Usual care	Red	Green	Red	Yellow	Green	Red
Cho, 2020	App only	Education	Green	Yellow	Red	Green	Yellow	Red
Cho, 2020	App + personalized coaching	Education	Green	Yellow	Red	Green	Yellow	Red
Oh, 2015	Mobile phone	Standard care	Yellow	Yellow	Red	Yellow	Green	Red
Thomas, 2017	Weight Watchers Online	Online newsletter	Green	Green	Red	Green	Red	Red
Thomas, 2017	Weight Watchers Online+	Online newsletter	Green	Green	Red	Green	Red	Red
Ferrante, 2020	SparkPeople + tracker	Waitlist active control group	Green	Yellow	Yellow	Green	Yellow	Red
Dunn, 2019	Photo diet self-monitoring app	Calorie-tracking self-monitoring app	Green	Green	Red	Green	Yellow	Red
Eisenhauer, 2021	Enhanced mobile technology	Basic mobile technology	Green	Yellow	Yellow	Green	Green	Yellow
Ross, 2016	Technology-based self-monitoring tools	Standard self-monitoring tools	Green	Green	Green	Green	Green	Green
Ross, 2016	Technology tools + phone intervention	Standard self-monitoring tools	Green	Green	Green	Green	Green	Green
Falkenhein, 2021	Keto (biofeedback)	Calorie-restricted	Green	Green	Green	Red	Yellow	Red
Bennett, 2019	App with feedback + clinical intervention	Usual care	Green	Green	Green	Green	Yellow	Yellow
Burke, 2022	App for feedback + control group	Active comparator	Green	Red	Green	Red	Green	Red
Hutchesson, 2018	Be positive be healthe (BPBH)	Waitlist	Green	Green	Yellow	Red	Green	Red
Lim, 2020	nBuddy + dietian	Standard care	Green	Yellow	Red	Yellow	Green	Red
Lim, 2021	nBuddy + dietian	Standard care	Green	Green	Yellow	Green	Green	Yellow
Spring, 2013	Mobile+	Standard care	Green	Yellow	Red	Green	Green	Red
Turner-McGrievy, 2017	App	Bite device	Green	Green	Red	Green	Yellow	Red
Vaz, 2021	App + visits	Visits + waitlist	Green	Green	Green	Green	Green	Green
Block, 2015	Alive-PD	Waitlist	Green	Green	Green	Green	Yellow	Yellow
Carter, 2013	App	Website	Green	Red	Red	Green	Green	Red
Carter, 2013	App	Paper diary	Green	Red	Red	Green	Green	Red
Spring, 2017	ENGAGED	Standard care	Green	Green	Green	Green	Green	Green
Spring, 2017	ENGAGED	Self-guided	Green	Green	Red	Green	Green	Red
Thomas, 2019	Smartphone	Group sessions	Green	Green	Green	Green	Green	Green
Thomas, 2019	Smartphone	Weight loss information	Green	Green	Green	Green	Green	Green

Note. Columns labelled “1” to “5” respectively indicate assessments for the randomisation process, deviations from the intended interventions, missing outcome data, measurement of the outcome, and selection of reported results. Column “F” shows the final score. Green = low risk of bias, yellow = some concerns, red = high risk of bias. Cochrane risk-of-bias tool for randomised trials (RoB 2) was used.

Figure S8

Involvement of Medical Worker as a Moderator (6-month Follow-up)

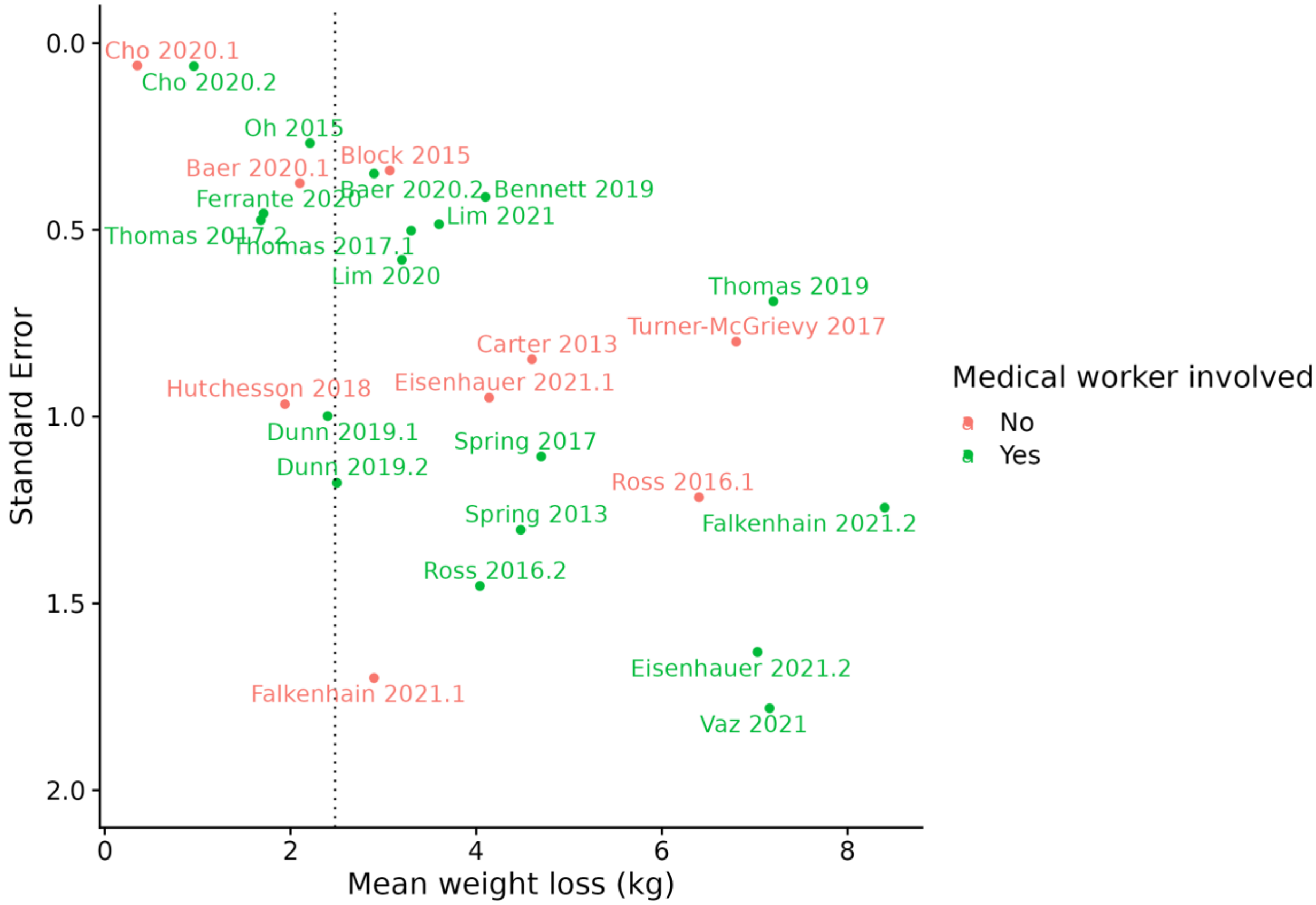

Note. The dashed line indicates the bias-corrected average for weight loss at 6 months from a three-level meta-analysis. No significant moderation effect was found.

Figure S9

Automatic Feedback Reports as a Moderator (6-month Follow-up)

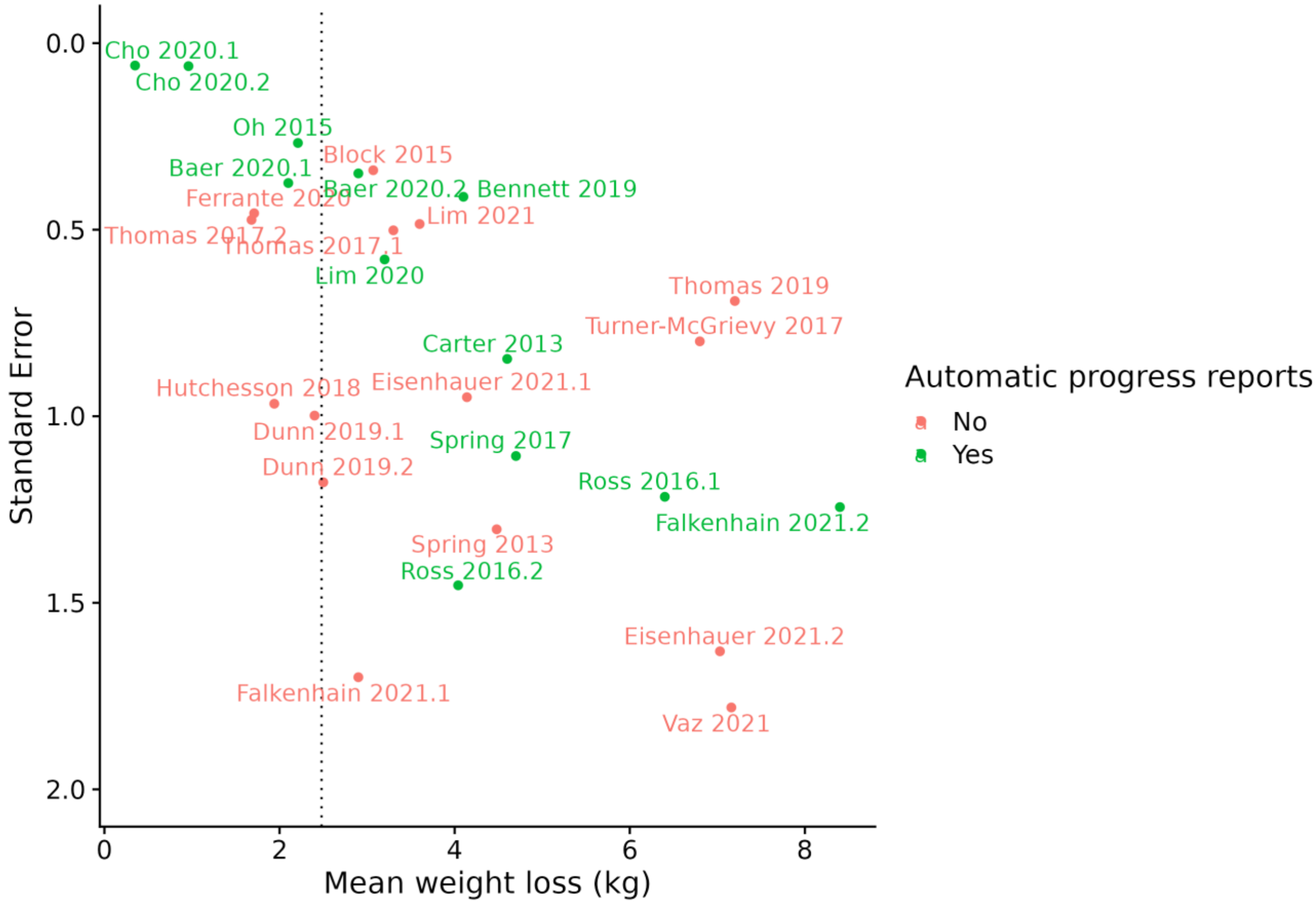

Note. The dashed line indicates the bias-corrected average for weight loss at 6 months from a three-level meta-analysis. No significant moderation effect was found.

Figure S10

Type of Calorie Counting as a Moderator (6-month Follow-up)

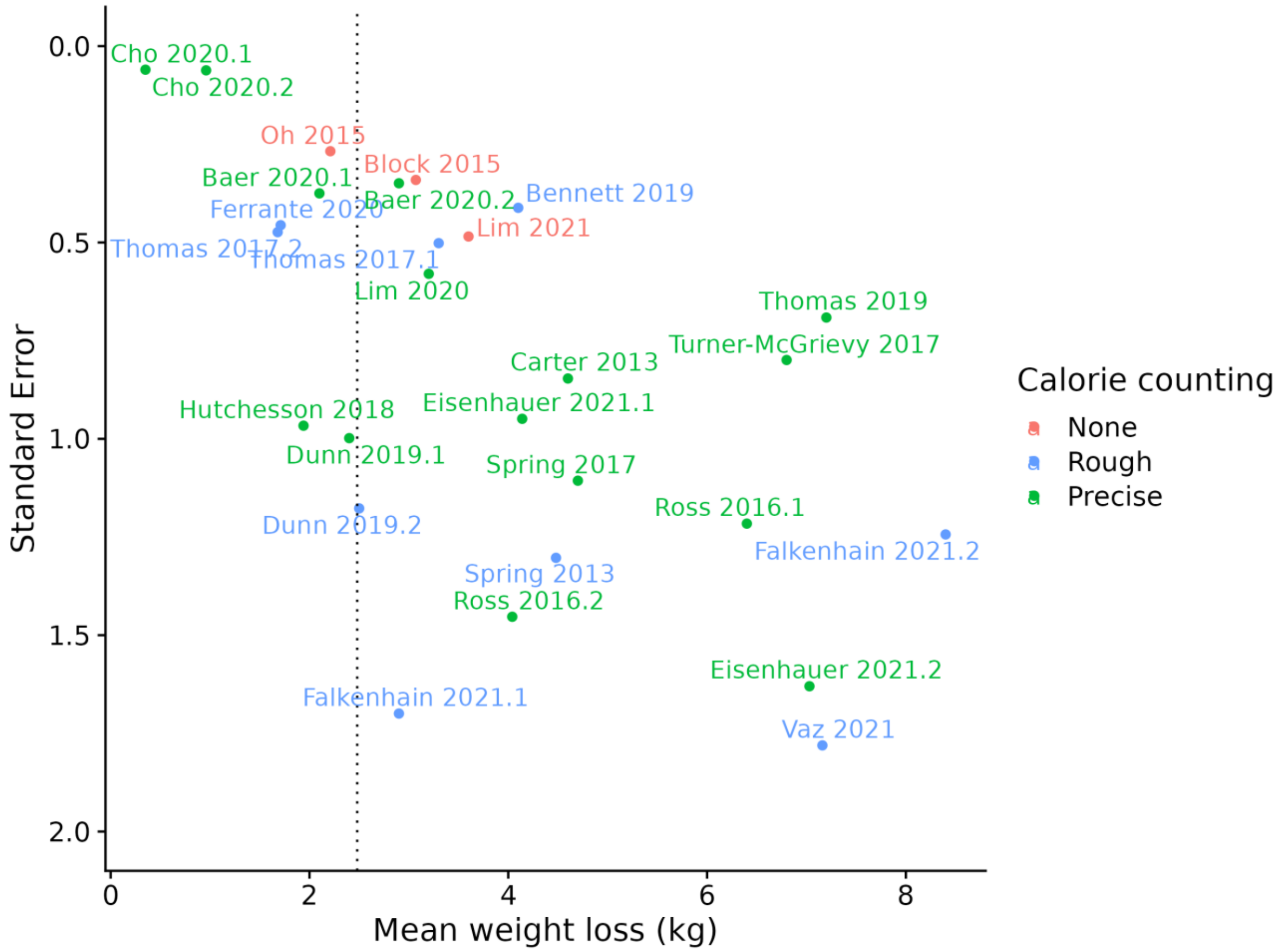

Note. The dashed line indicates the bias-corrected average for weight loss at 6 months from a three-level meta-analysis. No significant moderation effect was found.

Figure S11

Prescribed Menus or Recipes as a Moderator (6-month Follow-up)

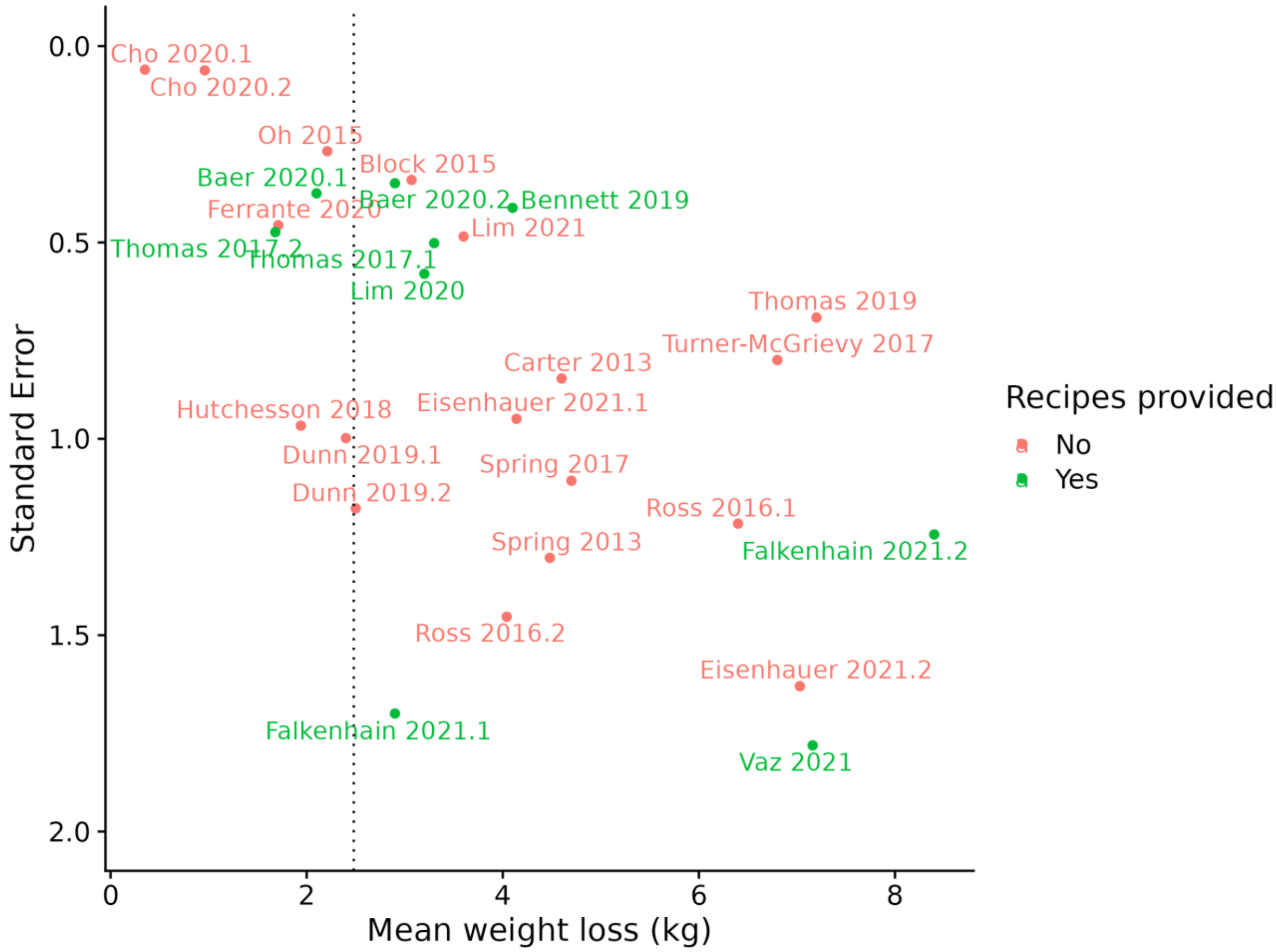

Note. The dashed line indicates the bias-corrected average for weight loss at 6 months from a three-level meta-analysis. No significant moderation effect was found.

Figure S12

Inclusion of Articles about Weight-loss as a Moderator (6-month Follow-up)

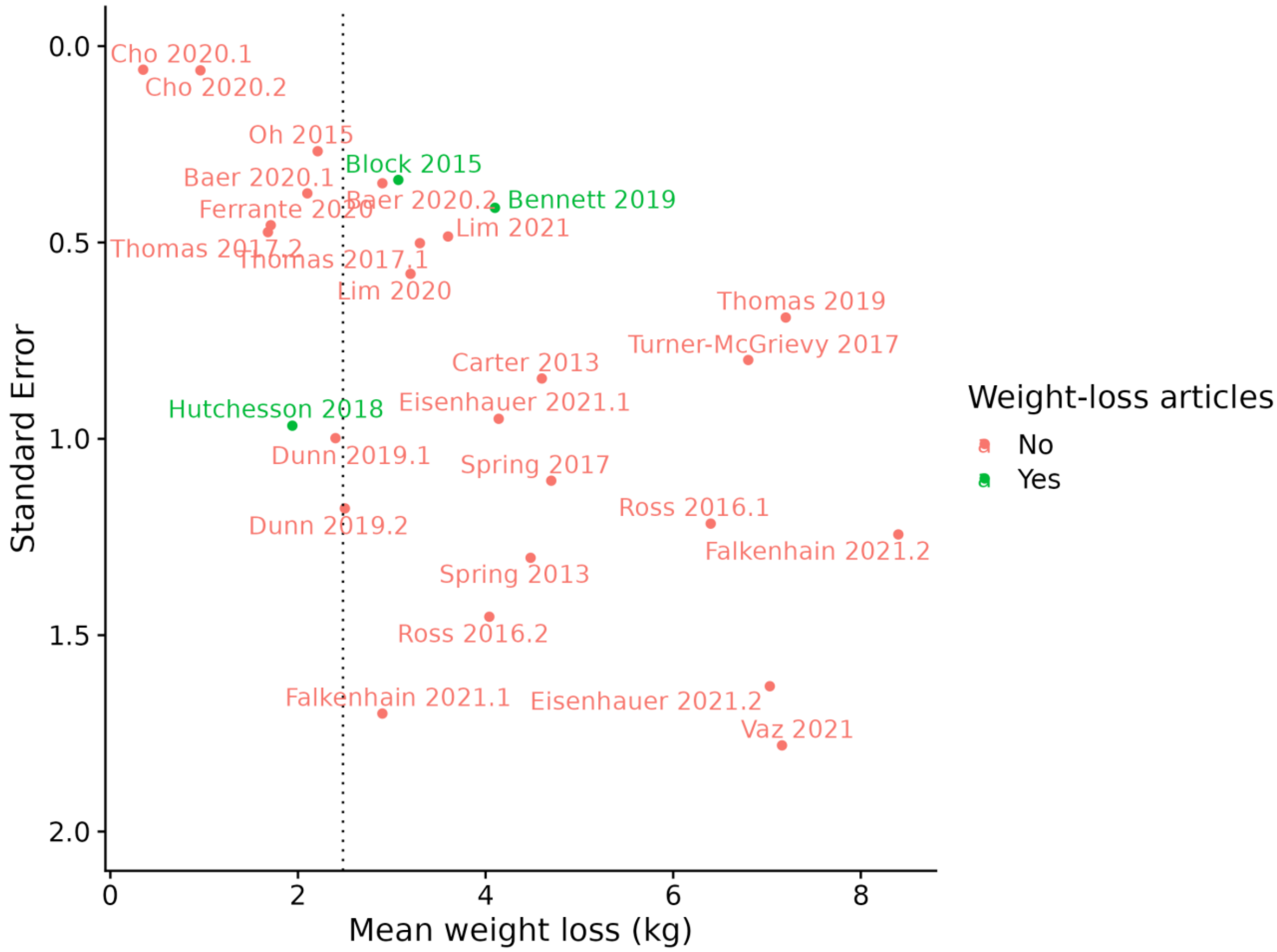

Note. The dashed line indicates the bias-corrected average for weight loss at 6 months from a three-level meta-analysis. No significant moderation effect was found.

Figure S13

Inclusion of Weight-loss Podcasts or Videos as a Moderator (6-month Follow-up)

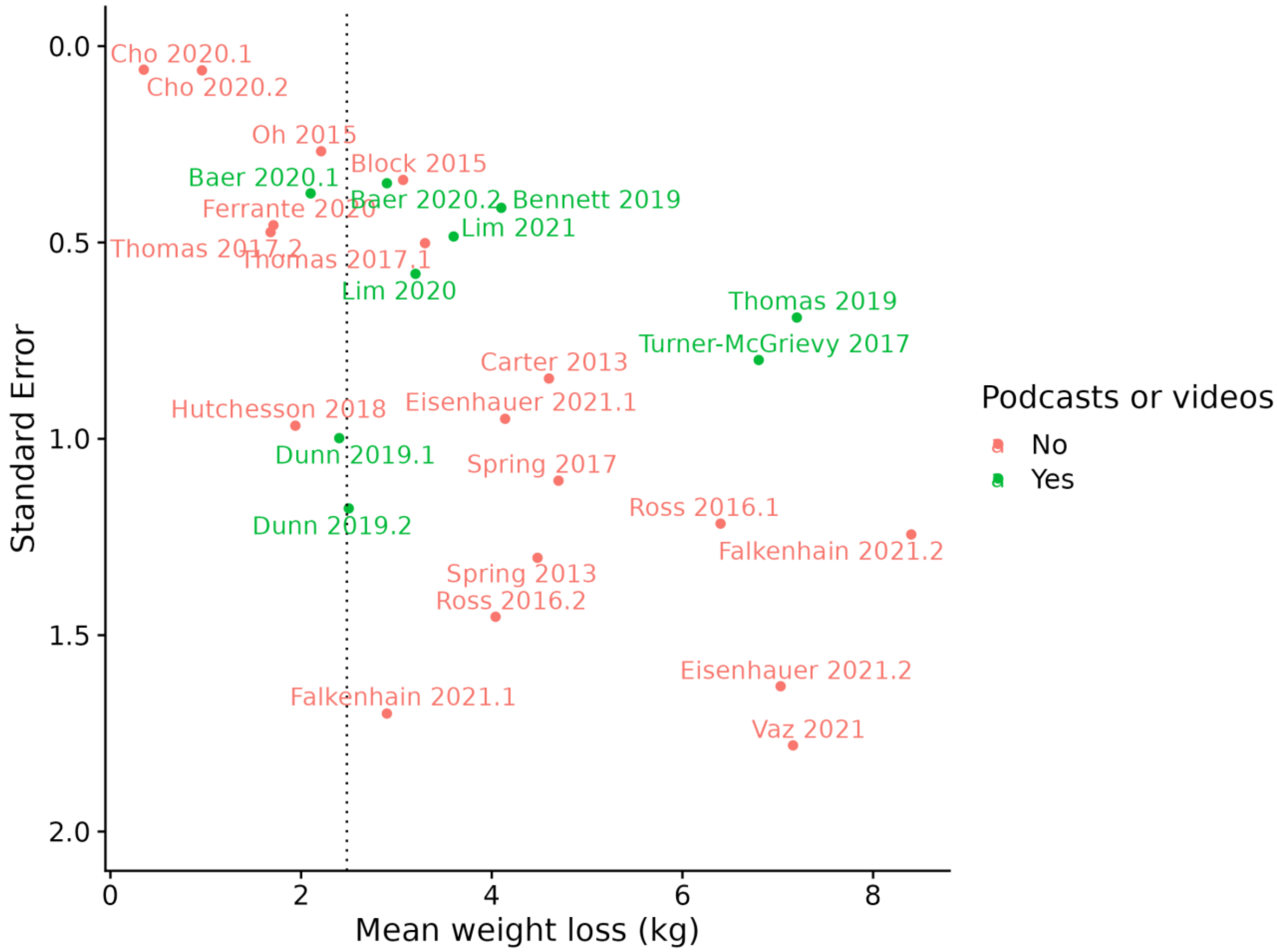

Note. The dashed line indicates the bias-corrected average for weight loss at 6 months from a three-level meta-analysis. No significant moderation effect was found.

Figure S14

Social Support Type as a Moderator (6-month Follow-up)

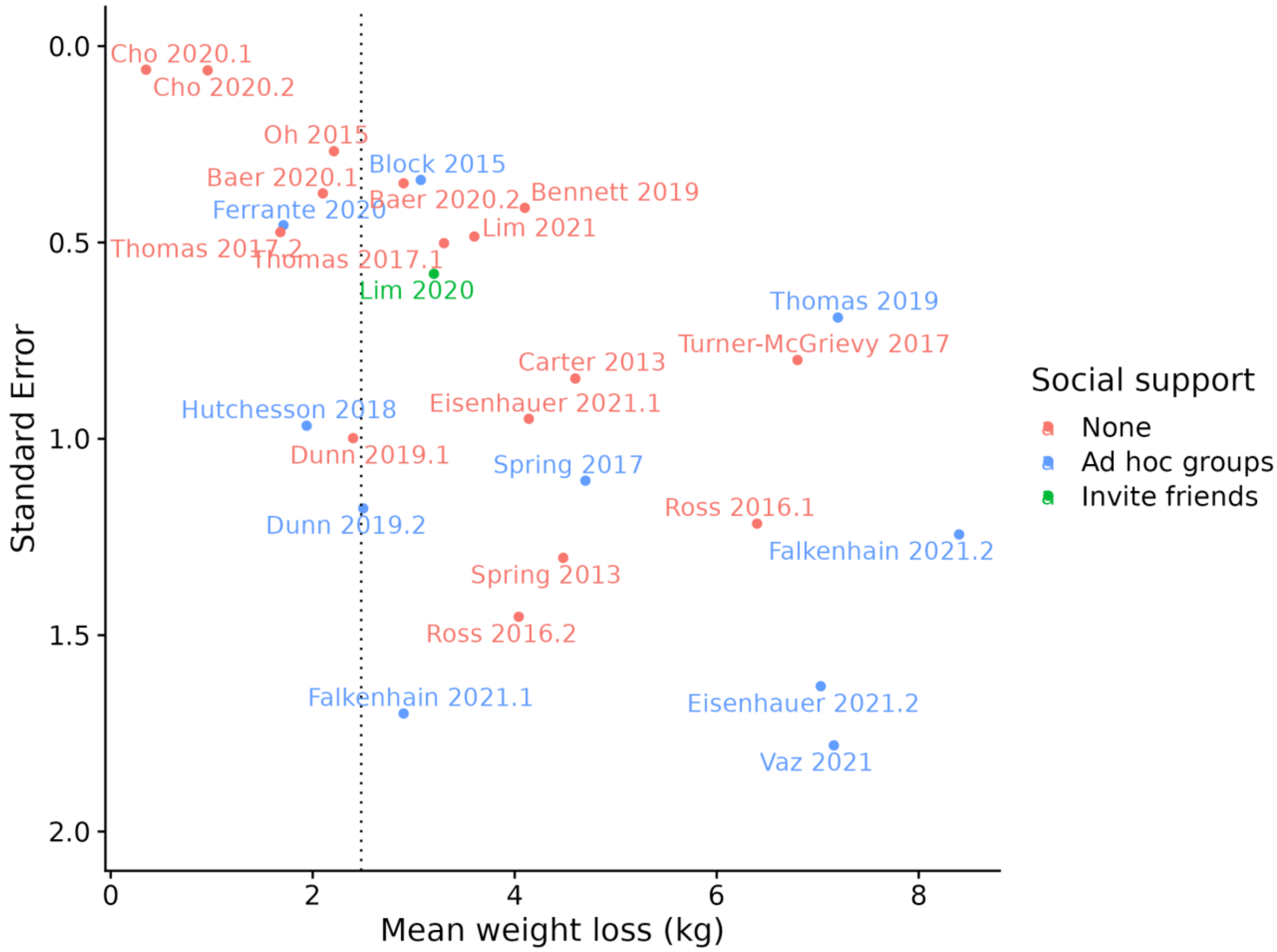

Note. The dashed line indicates the bias-corrected average for weight loss at 6 months from a three-level meta-analysis. No significant moderation effect was found.

Figure S15

Trial Duration as a Moderator (6-month Follow-up)

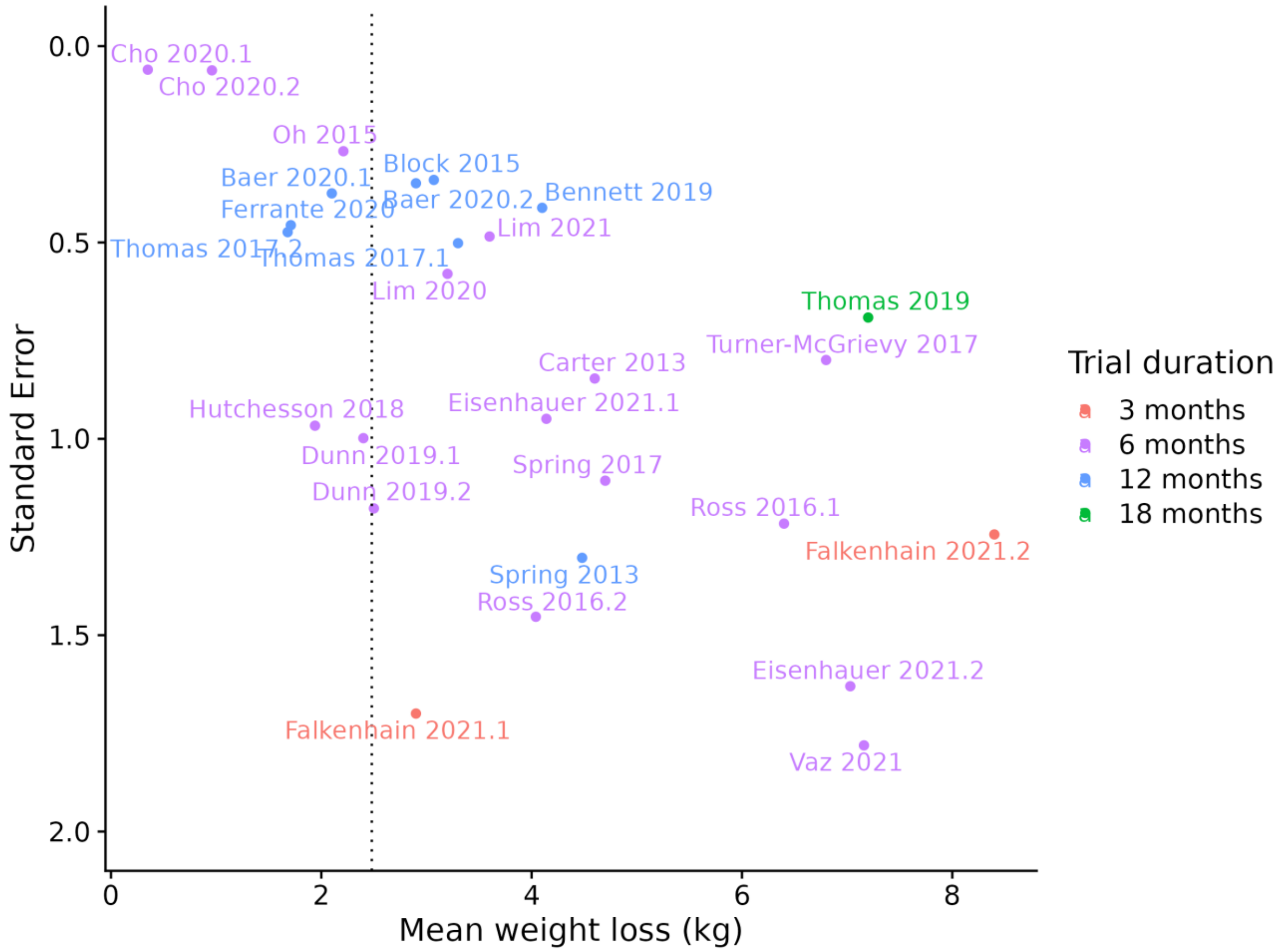

Note. The dashed line indicates the bias-corrected average for weight loss at 6 months from a three-level meta-analysis. No significant moderation effect was found.

Figure S16

Enrolment Type as a Moderator (6-month Follow-up)

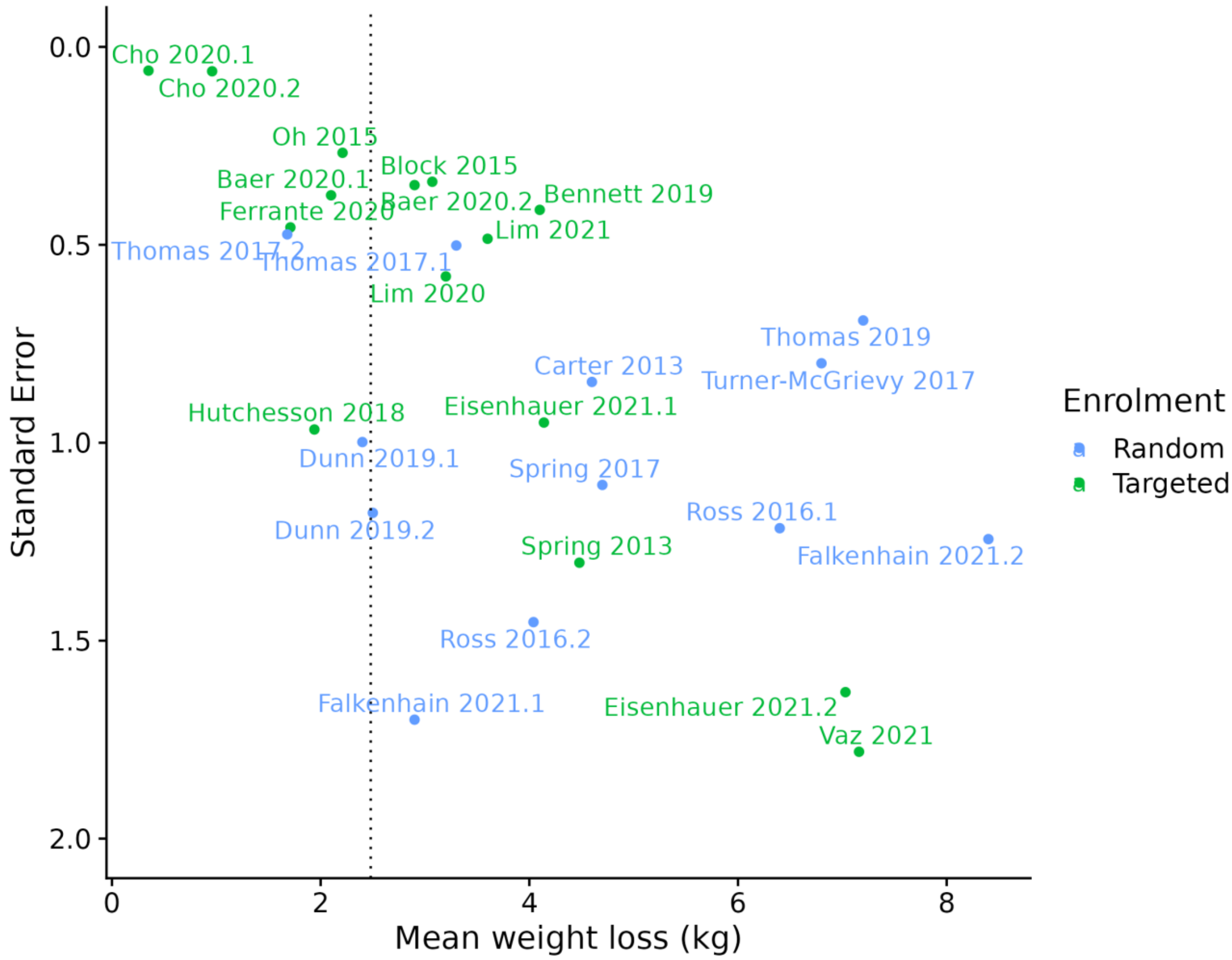

Note. The dashed line indicates the bias-corrected average for weight loss at 6 months from a three-level meta-analysis. No significant moderation effect was found.

Figure S17

Mean BMI at Baseline as a Moderator (6-month Follow-up)

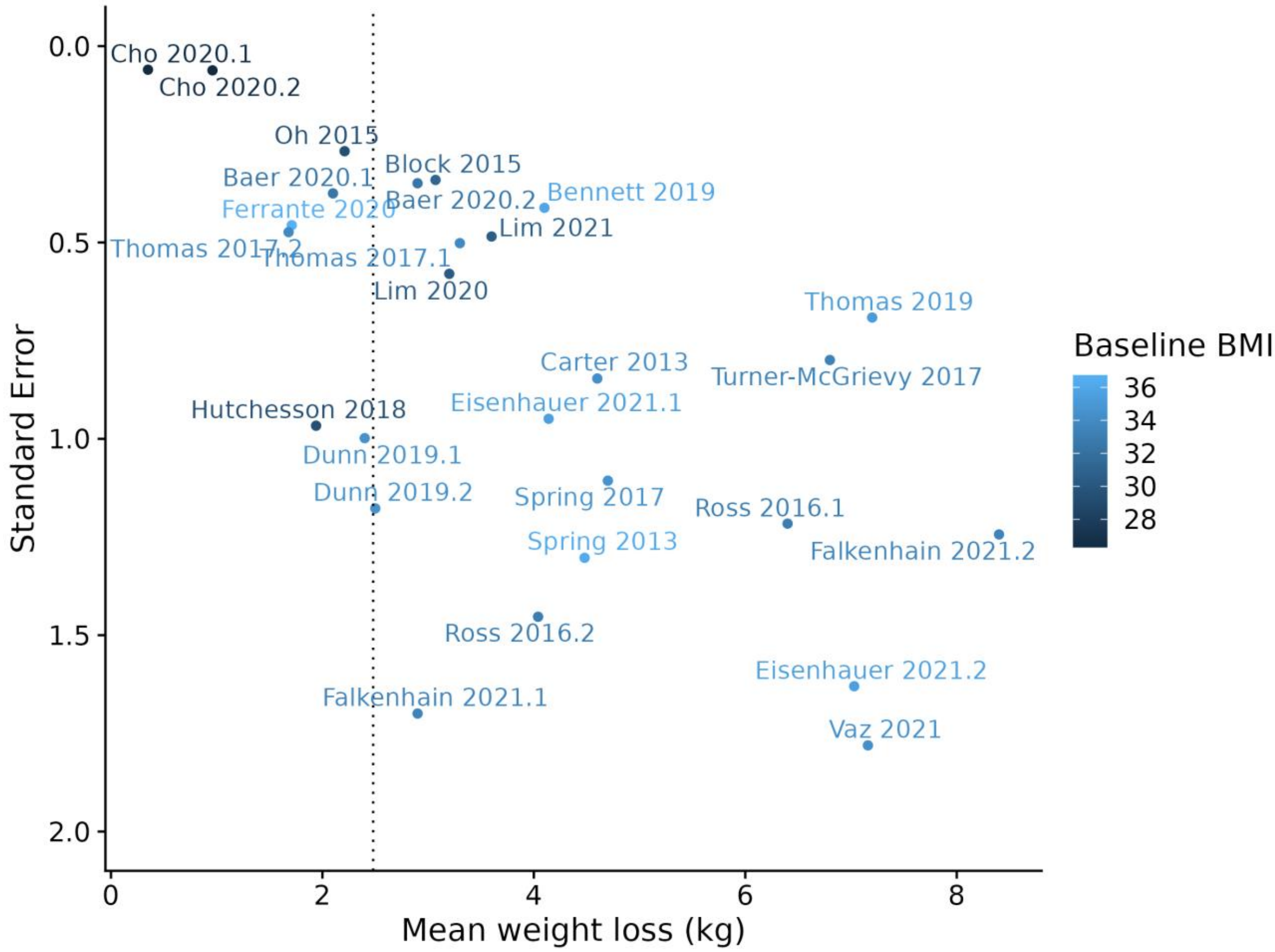

Note. The dashed line indicates the bias-corrected average for weight loss at 6 months from a three-level meta-analysis. No significant moderation effect was found.

Figure S18

Percent Female as a Moderator (6-month Follow-up)

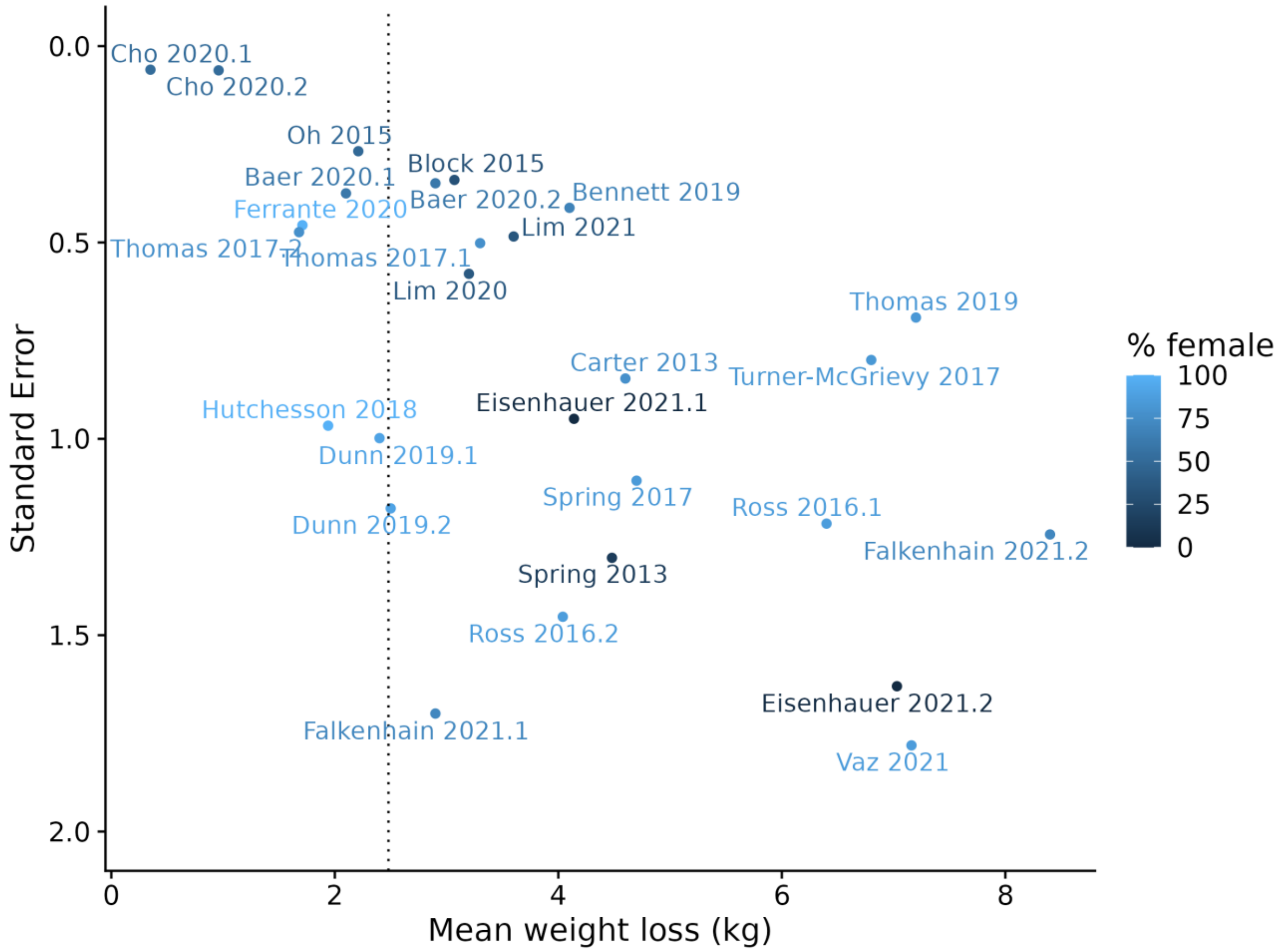

Note. The dashed line indicates the bias-corrected average for weight loss at 6 months from a three-level meta-analysis. No significant moderation effect was found.

Figure S19

Mean Age as a Moderator (6-month Follow-up)

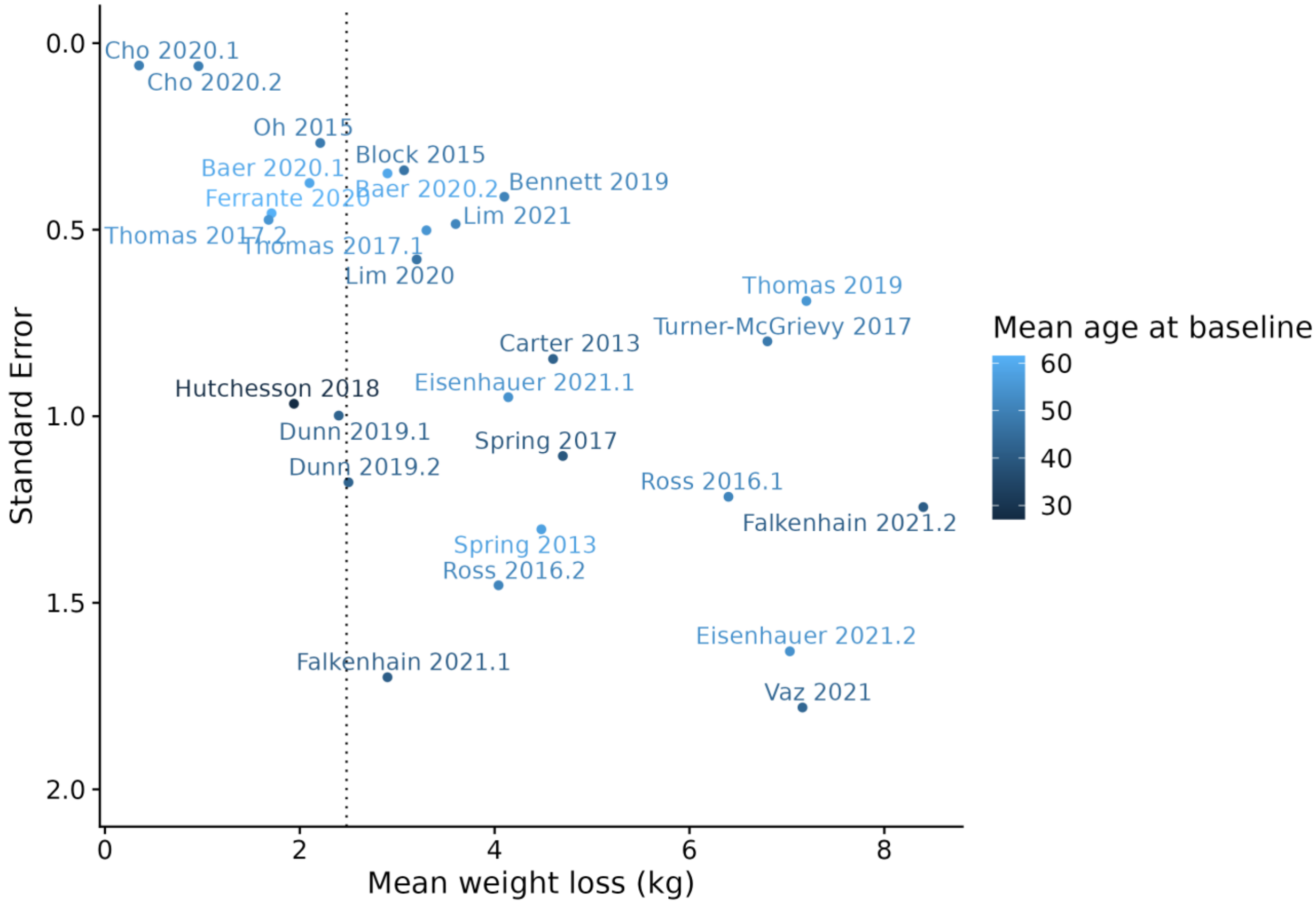

Note. The dashed line indicates the bias-corrected average for weight loss at 6 months from a three-level meta-analysis. No significant moderation effect was found.

Figure S20

Retention Rate as a Moderator (6-month Follow-up)

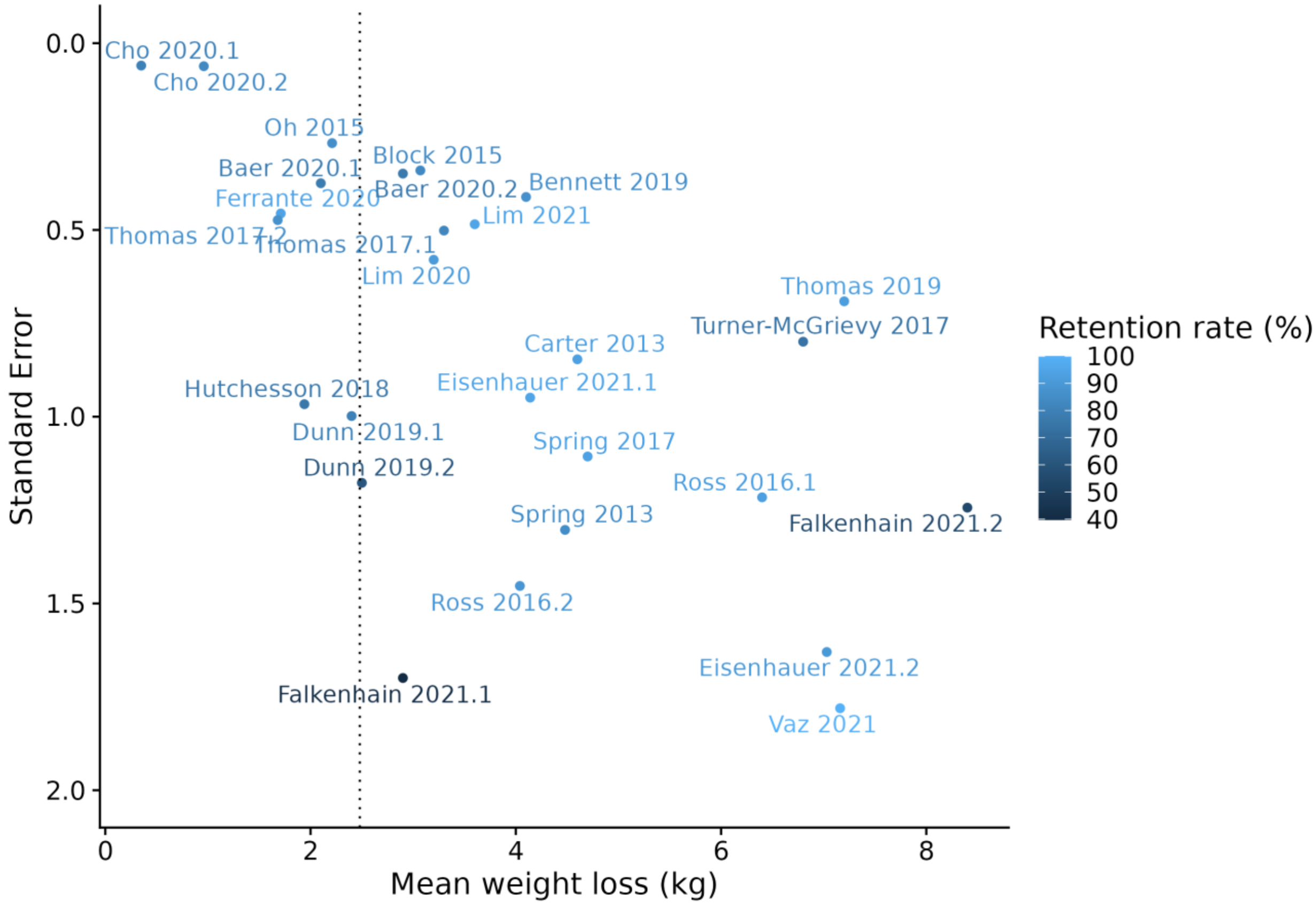

Note. The dashed line indicates the bias-corrected average for weight loss at 6 months from a three-level meta-analysis. No significant moderation effect was found.

Figure S21

Study Quality as a Moderator (6-month Follow-up)

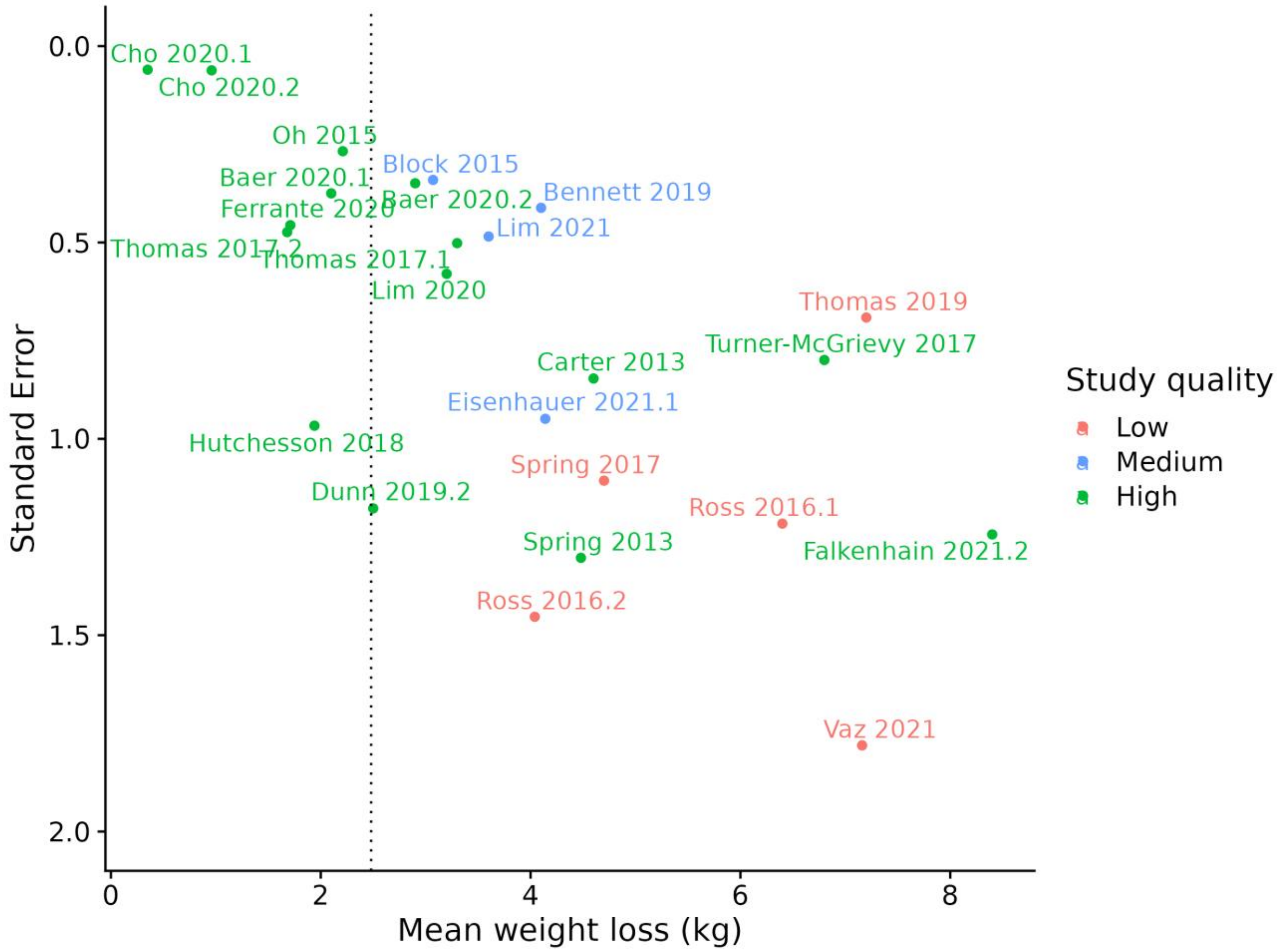

Note. The dashed line indicates the bias-corrected average for weight loss at 6 months from a three-level meta-analysis. No significant moderation effect was found.
